# Bis(*N*‐picolinamido)cobalt(II) Complexes Display Antifungal Activity toward *Candida albicans* and *Aspergillus fumigatus*

**DOI:** 10.1002/cmdc.202100159

**Published:** 2021-07-29

**Authors:** Laura H. D. Ghandhi, Stefan Bidula, Christopher M. Pask, Rianne M. Lord, Patrick C. McGowan

**Affiliations:** ^1^ School of Chemistry University of Leeds Woodhouse Lane Leeds LS2 9JT UK; ^2^ School of Chemistry University of East Anglia Norwich Research Park Norwich NR4 7JT UK; ^3^ School of Biological Sciences University of East Anglia Norwich Research Park Norwich NR4 7JT UK

**Keywords:** *Aspergillus fumigatus*, Bioinorganic Chemistry, *Candida albicans*, Cobalt Complexes, Picolinamide ligands

## Abstract

This report highlights the synthesis and characterization of ten new bis(*N*‐picolinamido)cobalt(II) complexes of the type [(L)_2_CoX_2_]^0/2+^, whereby L=*N*‐picolinamide ligand and X=diisothiocyanato (−NCS), dichlorido (−Cl) or diaqua (−OH_2_) ligands. Single crystal X‐ray (SC‐XRD) analysis for nine of the structures are reported and confirm the picolinamide ligand is bound to the Co(II) center through a neutral *N,O* binding mode. With the addition of powder X‐ray diffraction (PXRD), we have confirmed the *cis* and *trans* ligand arrangements of each complex. All complexes were screened against several fungal species and show increased antifungal activity. Notably, these complexes had significant activity against strains of *Candida albicans* and *Aspergillus fumigatus*, with several compounds exhibiting growth inhibition of >80 %, and onecompound inhibiting *Aspergillus fumigatus* hyphal growth by >90 %. Conversely, no antifungal activity was exhibited toward *Cryptococcus neoformans* and no cytotoxicity towards mammalian cell lines.

## Graphical abstract

Bis(*N*‐picolinamide)cobalt(II) complexes containing *trans* isothiocyanate ligands are reported to have significantly enhanced activity toward fungal species, when compared the *cis* complexes. They display >80 % growth inhibition of fungal strains and no reported toxicity toward bacterial species or mammalian cells.

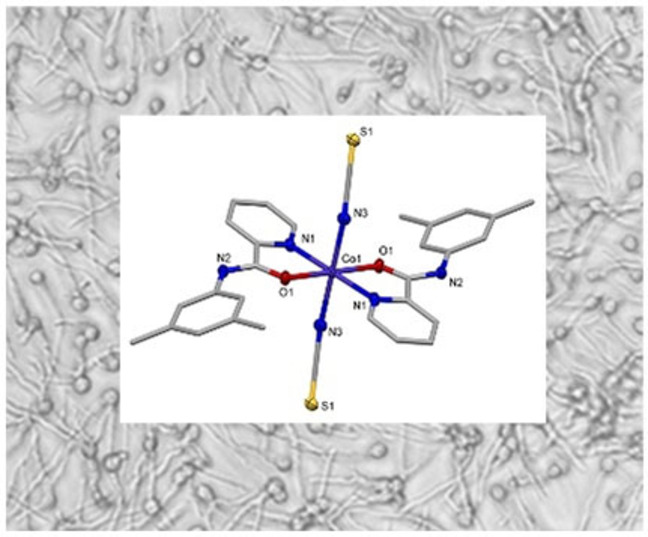

## Introduction

It is estimated that >1.5 million deaths can be attributed to fungal infections annually.^[1]^ The incidence of fungal infections is increasing and these infections are often associated with significant mortality rates in excess of 40 %, even with optimal treatment.[^1^, ^2^] This is partly due to the increased number of patients with HIV, those undergoing hematopoietic or whole organ transplantation, or receiving aggressive chemotherapy, for which opportunistic fungal or bacterial infections are particularly worrisome.^[3]^ The recent emergence of naturally drug resistant species, such as *Candida auris* and *Lomentospora prolificans*, or acquisition of drug resistance in the common fungal pathogens *Candida albicans* (*C. albicans*), *Cryptococcus neoformans* (*C. neoformans*) and *Aspergillus fumigatus* (*A. fumigatus*) only seeks to worsen the prognosis.[^4^, ^5^, ^6^, ^7^] In the USA, *C. albicans* is now the fourth most common iatrogenic bloodstream infection^[8]^ and in developing healthcare systems, infection with *Cryptococcus* spp. causes more deaths in HIV patients than tuberculosis.^[9]^ Furthermore, inhalation of fungi from *Aspergillus* spp. cause pathology in >14 million people and >200,000 deaths annually.^[1]^ There are currently only four main classes of antifungal drugs routinely utilized in the clinic: polyenes, azoles, allylamines and echinocandins. Thus, there is a crucial requirement for the development of new antifungal drugs.^[3]^

Despite the requirement for new antifungal drugs, there has been relatively little investigation into the potential of organometallic or coordination complexes to treat fungal infections. The nature of the metal ion, the geometry, oxidation states, hydrophobicity, and surrounding ligand environments all play an important role in improving the complex's activity. Moreover, it is well‐documented that the steric properties of the binding ligands effect the drugs pharmacokinetic properties.^[10]^ Therefore, the antimicrobial properties of metal complexes cannot be ascribed to chelation alone, and is a balance of several other contributions.

**Figure 1:**
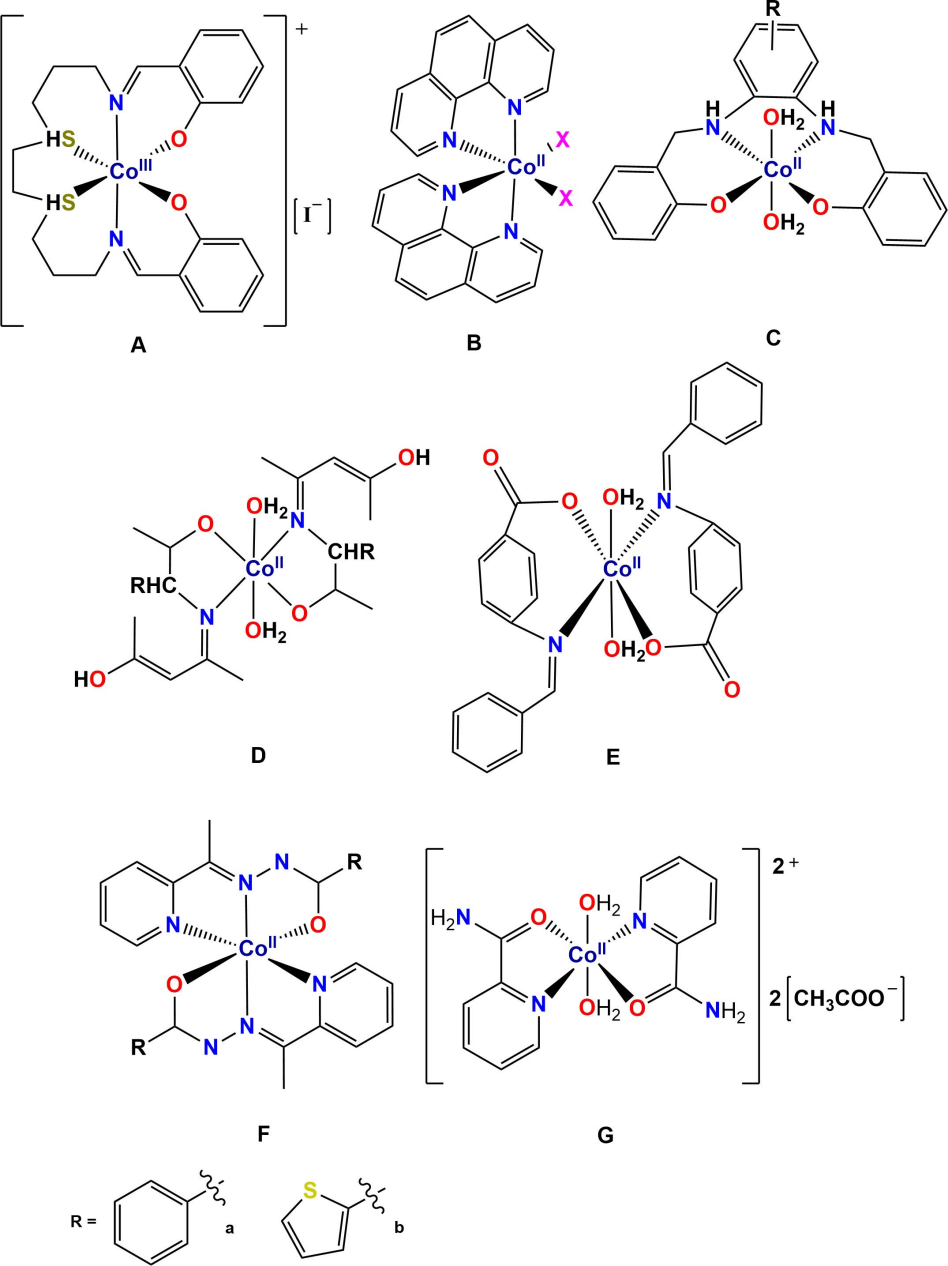
A range of cobalt complexes exhibiting anti‐microbial activity.

To date, few reports have been published which exclusively examine the antifungal activity of cobalt complexes. Cobalt plays a significant role in biological processes, as it is essential for metabolism, formation of red blood cells, maintenance of normal brain and nerve function and importantly it is found in vitamin B12.^[11]^ The latter contains a cobalt centre in the +1 oxidation state, however, synthetically, this metal is more commonly found in the +2 and +3 oxidation states. The first biological assays of cobalt were conducted in 1952 by Dwyer and co‐workers,^[12]^ and over the last decade there has been a surge in interest of cobalt coordination complexes as anticancer,[^11^, ^13^, ^14^, ^15^, ^16^] antimalarial,[^17^, ^18^] antibacterial[^19^, ^20^] and antifungal agents.[^21^, ^22^, ^23^, ^24^, ^25^, ^26^] In particular, 1,10‐bis(salicylideneamino)‐4,7‐dithiadecane cobalt(III) iodide (Figure 1A) was screened in mice and exhibited low systemic toxicity (LD_50_=75 mg kg^−1^), bacteriostatic and bactericidal activity against *Escherichia coli* (*E. Coli*) and *Staphylococcus haemolyticus* (μM range).^[12]^ Dwyer and co‐workers went on to screen a range of active Co(II) complexes containing phenanthroline ligands, which have been further adapted by several other research groups. All have highlighted complexes with moderate to good antimicrobial activity (e. g. Figure 1B).[^21^, ^27^, ^28^] Schiff‐base complexes with salan ligands (e. g. Figure 1C),[^22^, ^29^] amino acids (e. g. Figure 1D),^[30]^ and benzoic acids (e. g. Figure 1E),^[31]^ have also shown promising activity against a range of fungal species, with the majority of this research being conducted by Chohan and co‐workers.[^23^, ^24^, ^30^, ^32^, ^33^, ^34^] Other ligands which have been investigated, include; azoles,^[32]^ cephalexins,^[23]^ sulfonamides,^[33]^ dithiones,^[34]^ and thiolenes,^[24]^ with complexes exhibiting a wide range of antimicrobial activities when bound to other transition metals.

In 2012, Sathyadevi and co‐workers studied two Co(II)‐hydrazone complexes (Figure 1F, R=phenyl (1F‐a) or thiophenyl (1F‐b)),^[25]^ and reported that both complexes interact with Bovine Serum Albumin (BSA), with binding constants of 6.5×10^4^ M^−1^ (1F‐a) and 3.4×10^5^ M^−1^ (1F‐b), and the complexes display the ability to scavenge hydroxyl radicals. Additionally, these complexes were able to scavenge nitric oxide radicals, with antioxidant activity of the complexes ∼5.8× higher than the free ligand. Importantly, they demonstrated a greater capacity to prevent oxidation deterioration of lipids (important in human diseases^[35]^), and exhibited significant antibacterial and antifungal activities against a range of bacterial and fungal species.

**Figure 2:**
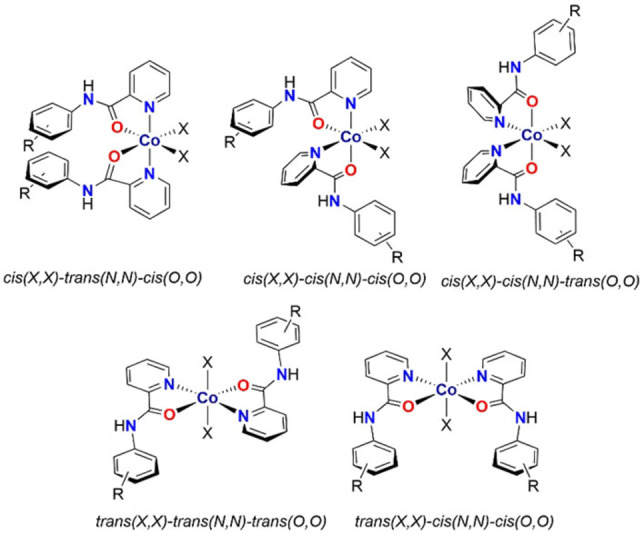
Potential isomers of [(L)_2_CoX_2_] (where L=a picolinamide ligand and X=any one electron ligand), with the *cis/trans* descriptors designated in the order: ancillary(*X,X*)‐picolinamide pyridyl ring (*N,N*)‐picolinamide amide group (*O,O*).

To date, the antifungal properties of bis(*N*‐picolinamido)cobalt(II) complexes have not yet been investigated, with previous literature only reporting the structural compositions of the complexes. The first study, in 1984, was conducted by Tsintsadze and co‐workers on [(L)_2_Co(H_2_O)_2_][(CH_3_COO)_2_] (Figure 1G, L=C_5_H_4_NC(O)NH_2_), in which they investigated how the position of the amide group from the pyridine ring influences the structure of the corresponding metal complexes.^[36]^ Structural data shows the dicationic octahedral Co(II) complex has two picolinamide ligands bound to the metal in a neutral *N,O* fashion, with two aqua ligands in a *trans* arrangement. Further studies with chloride or squarate acting as the counter‐ion confirmed this *trans* geometry.[^37^, ^38^] The corresponding Ni(II) and Zn(II) aqua complexes also display a *trans* geometry.^[39]^ Upon changing the axial aqua ligands to thiocyanate, a neutral cobalt complex is formed and the geometry switches to a *cis* arrangement, with the pyridyl rings remaining *trans* to one another.^[40]^ Density functional theory (DFT) calculations found that the lowest energy form of these complexes involves the picolinamide ligands in the *cis* geometry and the isothiocyanate ligands coordinating through the nitrogen (−N=C=S), as opposed to the sulfur atom (thiocyanato, −S−C≡N). The corresponding Ni(II) and Zn(II) isothiocyanato complexes also display the same *cis* geometry,^[23]^ however, the analogous Cu(II) complex contains the two ligands in a *trans* arrangement and coordinated to the through the sulfur atom. Generally, these types of bis(*N*‐picolinamido)metal complexes can exist as different isomers (Figure 2), with the *cis(X,X)‐trans(N,N)‐cis(O,O)* and *cis(X,X)‐cis(N,N)‐trans(O,O)* (where X can be any one electron or two electron ligand; the latter changing the formal charge of the complex) also existing as enantiomers. Therefore, it is important to identify the isomers, so their therapeutic activities can be correctly determined, however, this has not yet been reported for bis(*N*‐picolinamido)cobalt(II) complexes.

Herein we report the synthesis and evaluation of a library of new bis(*N*‐picolinamido)cobalt(II) complexes. The picolinamide ligands coordinate in a neutral *N,O* mode to the cobalt metal center and theX ligands (X=diisothiocyanato, dichlorido or diaqua) exist in either a *cis* or *trans* arrangement, which was confirmed by single crystal X‐ray diffraction (SC‐XRD). This technique also confirmed that in all cases, the isothiocyanato ligand is bound to the central cobalt through the nitrogen. Additionally, the cytotoxicity of all complexes has been determined through screening against a range of human cell lines, bacterial species and fungal species.

## Results and Discussion

**Scheme 1:**
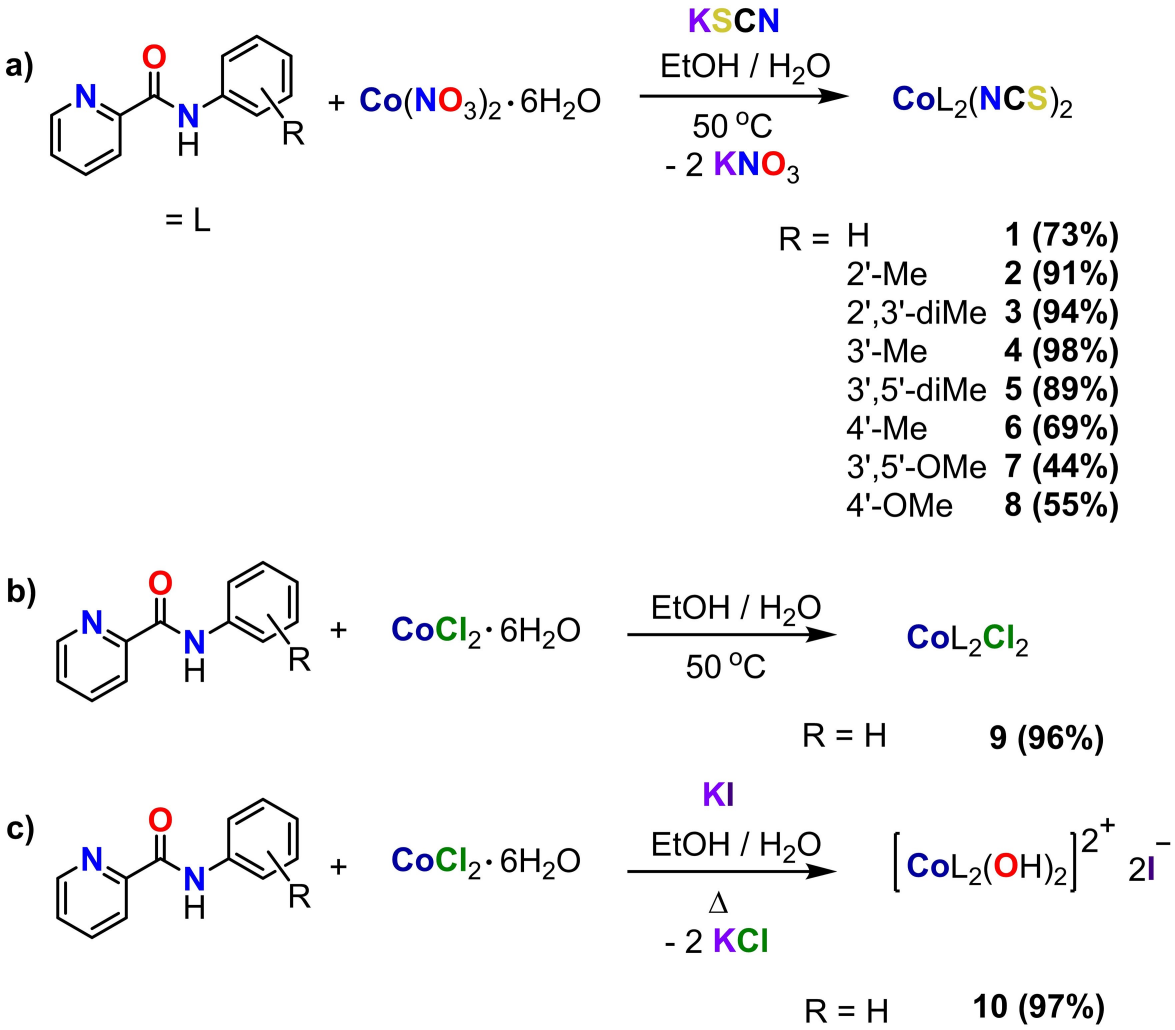
Synthetic routes for **a)** bis(*N*‐picolinamido)cobalt(II) diisothiocyanato complexes **1**–**8**, **b)** bis(*N*‐picolinamido)cobalt(II) dichlorido complex **9** and **c)** bis(*N*‐picolinamido)cobalt(II) diaqua complex **10**.

**Synthesis of Co(II) complexes**: The *N*‐picolinamide ligands were synthesized *via* a known literature procedure from the condensation of picolinic acid with a functionalized aniline.^[41]^ All ligands have been previously reported and ^1^H NMR was used to confirm successful synthesis.[^42^, ^43^, ^44^, ^45^, ^46^, ^47^, ^48^, ^49^, ^50^] The bis(*N*‐picolinamido)cobalt(II) diisothiocyanato complexes, [(**L**)_2_Co(NCS)_2_] **1**–**8**, were synthesized by the heating (15 mins) of cobalt(II) nitrate hexahydrate (1 eq.) with a functionalized *N*‐picolinamide ligand (**L**, 2 eq.), followed by stirring with potassium thiocyanate (2 eq.) at room temperature for 2 h. All complexes were isolated as pink‐purple precipitates in moderate to good yields (44–98 %, Scheme 1a).^[40]^ The Co‐dichlorido analogue, complex **9**, was prepared by heating/stirring cobalt(II) chloride (1 eq.) with a functionalized picolinamide ligand (2 eq.) for 2 h, to yield a pale orange precipitate (98 %, Scheme 1b). The synthesis of the dichlorido analogue was only successful when no electron withdrawing groups were present on the aryl ring of the ligand, and this may be explained by the increased acidity of the amide proton when electron withdrawing groups are present, hence hindering the *N,O* coordination to the metal center. Furthermore, the dichlorido analogue was reacted with potassium iodide (2 eq.) and heated/stirred for 2 h, to form the dicationic Co‐diaqua complex **10** as an orange precipitate (97 %, Scheme 1c).

All complexes exhibited broad ^1^H NMR spectra due to the paramagnetic nature of the complexes (Co(II), d^7^), however the positioning of the peaks remains unchanged compared to the free ligands allowing successful assignment. The IR spectra of complexes show a broad NH stretch at 3200–3300 cm^−1^, a strong isothiocyanato CN stretch at 2070–2100 cm^−1^ (**1**–**8**) and a strong CO stretch at 1610–1635 cm^−1^, and these peaks are shifted to lower wavenumbers when compared to the free ligands. The appearance of the CN stretch is characteristic of the formation of diisothiocyanato complexes and was used to confirm successful synthesis. The UV‐vis spectra show intense ligand‐based π‐π* absorbance at ∼200 nm, less intense charge‐transfer bands between 220–280 nm and weak d–d transitions at ∼640 nm (e. g. Figure S2).

**Figure 3:**
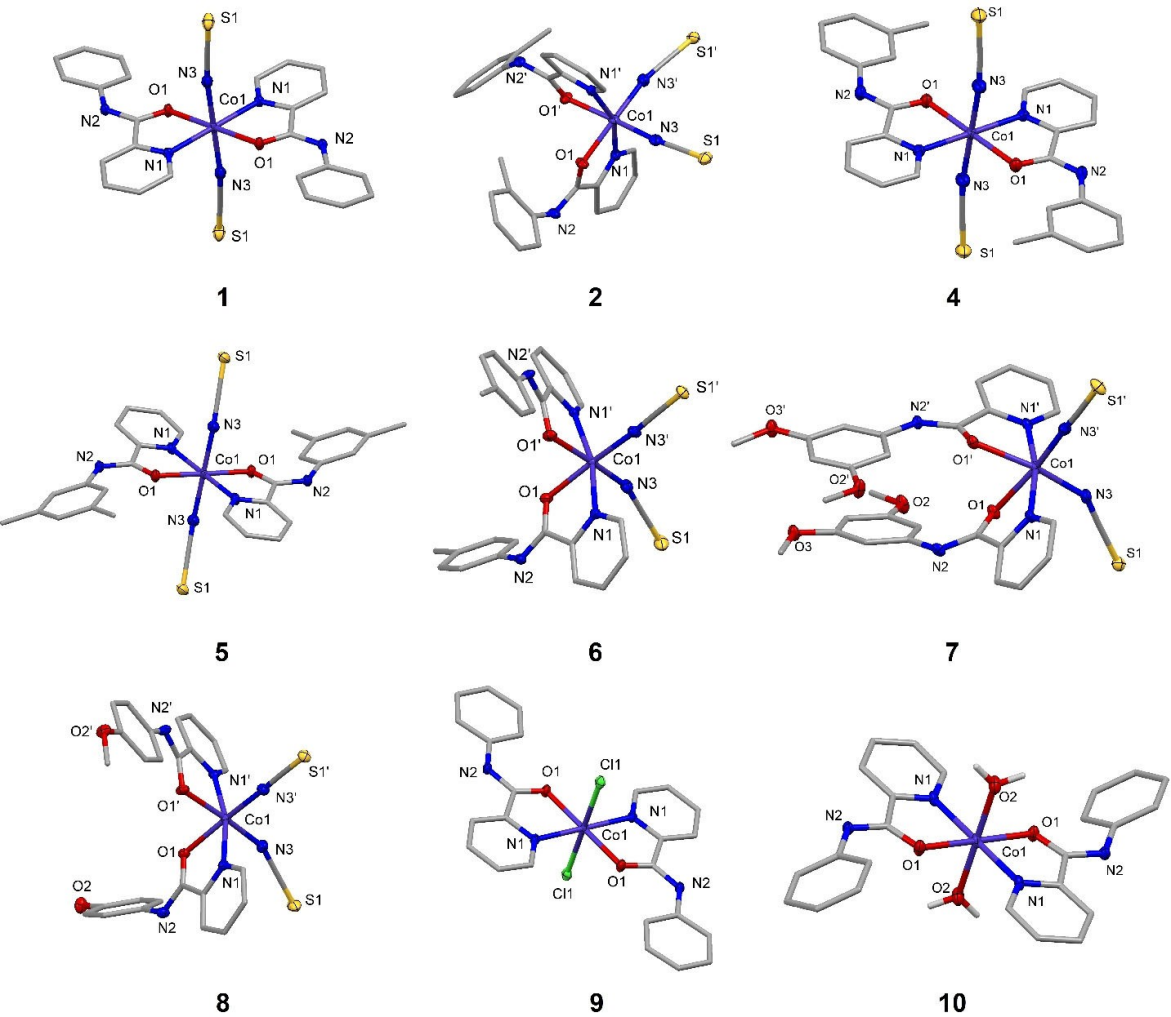
Molecular structures of complexes **1**, **2**, **4**–**10**. Displacement ellipsoids are at the 50 % probability level for heteroatoms only. Hydrogen atoms and counterions are omitted for clarity.

**Single‐crystal X‐ray diffraction (SC‐XRD)**. Single crystals suitable for SC‐XRD analysis were obtained for complexes **1**, **2**, **4**–**10** (Table S1–S2, CSD: 2065375–2065383) via slow evaporation of the reaction solvent. The molecular structures for these complexes are presented in Figure 3, with selected bond lengths and angles presented in Table 1 and Table S3, respectively. Single pink‐purple crystals were obtained for diisothiocyanato complexes **1**, **2** and **4**–**8**, with solutions performed in either a triclinic (*P*‐1: **1**, **5** and **7**) or monoclinic (*P*2_1_/*c*: **2** and **4**, *C*c: **6** and **8**) cell. On changing the ancillary diisothiocyanato ligands to either dichlorido (**9**) or di‐aqua (**10**), single orange crystals were obtained, with structural solutions performed in a monoclinic (*P*2_1_/*c*) cell. The crystal structures confirm that the two picolinamide ligands coordinate through the pyridyl nitrogen and amide oxygen, acting as neutral *N,O* ligand.[^36^, ^37^, ^40^] The axial isothiocyanato ligands coordinate to the Co(II) center through the nitrogen atom, as opposed to through the sulfur atom, and this result is consistent with the previous complexes and DFT calculations performed by Đaković and co‐workers.^[40]^

**Table 1:** Selected bond lengths (Å) of complexes **1**, **2**, **4**–**10**, where X=N (**1**–**8**), Cl (**9**) or O (**10**), s.u.s in parenthesis.

	Bond Lengths [Å]		
	Co(1)−N(1)/ Co(1)−N(1’)	Co(1)−O(1)/ Co(1)−O(1’)	Co(1)−X(1)/ Co(1)−X(2)
**1** ^[a]^	2.1064(15)	2.1021(12)	2.0828(17)
**2**	2.135(3)/ 2.141(3)	2.122(3)/ 2.138(3)	2.070(4)/ 2.048(4)
**4** ^[a]^	2.117(2)^[^	2.0934(17)	2.070(2)
**5** ^[a]^	2.113(2)	2.124(2)	2.060(3)
**6**	2.1064(15)	2.1021(12)	2.0828(17)
**7**	2.113(4)/2.123(4) 2.127(4)/2.112(4) 2.107(4)/2.115(4) 2.119(4)/2.104(4)	2.201(4)/2.206(4) 2.231(4)/2.194(4) 2.204(4)/2.189(4) 2.199(3)/2.195(4)	2.070(5)/2.046(5) 2.055(5)/2.061(5) 2.043(5)/2.095(5) 2.081(5)/2.042(5)
**8**	2.129(2)/ 2.102(2)	2.164(2)/ 2.144(2)	2.048(3)/ 2.081(3)
**9** ^[a]^	2.0887(18)	2.0937(15)	2.4462(7)
**10** ^[a]^	2.093(3)	2.046(2)	2.121(3)

[a] Symmetry generated.

The Co−N(pyridyl) bond lengths are in the range 2.0887(18)–2.141(3) Å and the Co−O(amide) bond lengths are in the range 2.0934(17)–2.230(4) Å. This is consistent with related high spin (HS) Co(II) compounds which have reported to have Co−N(pyridyl) bond lengths of 2.07–2.12 Å and Co−O(amide) bond lengths of 2.06–2.12 Å.[^36^, ^37^, ^40^] All complexes show a distorted octahedral geometry around the central Cot(II) center, with the relevant bond angles in the range 75.90(12)–99.52(14)°, deviating from the ideal 90° (Table S3). This distortion can be ascribed to the restrictions imposed by the bidentate *N*‐picolinamide ligand. The crystal structures highlight intramolecular H‐bonding between the O(amide) and the *ortho* Ar−CH of the same ligand, with bond distances of 2.83–2.86 Å, intermolecular H‐bonding between the S(isothiocyanate) and the neighboring N−H(amide), with bond distances of 3.32–3.61 Å, and π–π stacking between the aryl and pyridyl rings of neighboring molecules, with distances of 3.55–3.87 Å (e. g. Figure S1).

As previously stated, each bis(*N*‐picolinamido)cobalt(II) complex may form five potential (plus two enantiomers) isomers (Figure 2), however only the *trans‐trans‐trans* and *cis‐trans‐cis* isomers have been observed in our solid‐state analysis. Complexes **1**, **4**, **5**, **9** and **10** contain the two X ligands in a *trans* arrangement, whereas complexes **2** and **6**–**8** have the X ligands in a *cis* arrangement. In almost all cases, when the aryl ring is unsubstituted or substituted in the *meta* position, the X ligands are positioned in a *trans* arrangement; whereas substitution in the *ortho* or *para* positions on the aryl ring result in the corresponding *cis* complexes.

Of the reported bis(*N*‐picolinamido)cobalt(II) complexes, the X ligands are described to be in the *trans* position, though differences in *cis/trans* geometry have been noted with alterations in the axial ligand from aqua^[36]^ to isothiocyanato,^[40]^ and upon changing the metal ion from cobalt to copper.^[40]^ However, it appears that the nature of the axial ligand is an important factor. All previous studies have incorporated picolinamide ligands with no *N*‐aryl substitutions (similar to complex **1**) and thus this work presents the first study on the geometric effects of changing the picolinamide ligand substituents. This work implies that the effect of ligand substituents on geometry is greater than the effect of the type of axial ligand or the nature of the cobalt starting material. It is suggested that the geometries have differing thermodynamic stabilities depending upon the *N*‐aryl substitution, although the reason for these differences is unclear. The fact that altering the position of the same group, e. g. methyl, alters the geometry implies that steric effects are of greater importance than electronic effects.

**Figure 4:**
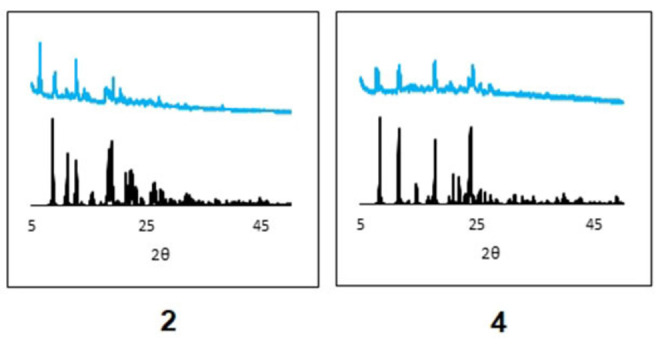
Powder X‐ray Diffraction (PXRD) patterns for complexes **2** (*cis*) and **4** (*trans*); **black**=simulated and **blue** **=**experimental.

**Powder X‐ray diffraction (PXRD)**. PXRD patterns of several complexes were recorded to allow comparison with the SC‐XRD data (Figure 4). In the solid state, complexes **1**, **4** and **5** adopt the *trans* geometry whereas complexes **2**, **6**, **7** and **8** adopt the *cis* geometry. All the *trans* complexes show good agreement between the experimental and the simulated powder diffraction patterns, with all of the high intensity peaks from the simulated pattern present at similar 2θ positions in the experimental patterns. This confirms that the bulk samples of these complexes consist of only the *trans* isomer. For the *cis* complexes, the high intensity peaks from the simulated pattern do appear in the experimental patterns, but some additional intense peaks are also present in the experimental diffraction patterns, which cannot be matched to the corresponding simulated pattern. This could be ascribed to the presence of a mixture of isomers in the bulk powder sample.[^48^, ^51^] If the observed differences are due to the presence of a mixture of isomers, as this is only observed for *cis* complexes, this may imply that the *trans* complex is the thermodynamic product and the *cis* complex (or mixture) is the kinetic product of the complex synthesis. These mixtures of *cis* isomers, and stability of the *trans* isomers has already been observed in our group, when *N*‐picolinamide ligands are bound to Ru(III)^[48]^ and Rh(III)^[51]^ metal centers.

**Magnetic properties**. Previous bis(picolinamido)cobalt(II) complexes have been reported to be high spin (HS) with μ_eff_=4.1 μ_B_.^[52]^ Our complexes exhibited poor solubility, therefore, the magnetic behavior was only studied for complex **2** in both solution and solid state. The solution state studies were conducted via the Evans NMR method, and complex **2** was found to have a magnetic moment 4.01 μ_B_ at 300 K in d_3_‐acetonitrile, consistent with a HS system. The magnetic moment as a function of temperature was also measured by a superconducting quantum interference device (SQUID), and 4.36 μ_B_ from 300–75 K before dropping at lower temperatures due to the effects of zero field splitting (Figure S5). This supported the solution state data and indicates a HS system.

**Cyclic voltammetry**. Co(II) complexes can exist as either low spin (LS) or HS, although octahedral Co(II) systems are more often HS. However, these d^7^ systems can have fast transitions between both spin states, known as spin crossover (SCO). SCO in Co(II) complexes is uncommon and often gradual or incomplete; however there are examples of ligand systems which display abrupt SCO with thermal hysteresis, these ligands tend to be terpyridine‐based systems.^[53]^ In terms of the spectrochemical series, our complexes have varying ancillary ligands, whereby X=diisothiocyanato (**1**–**8**), dichlorido (**9**) or diaqua (**10**), and so we have assessed if these difference in crystal field splitting (Δo) energies can affect the complexes spin properties.

In 2012, Harding and co‐workers showed Co(II)‐bipyridine acetylacetone complexes, with a single electron oxidation Epa_1_, a small reduction Epc_1_ and a larger reduction Epc_2_.^[54]^ They concluded the oxidation/reduction is accompanied with a structural and electronic rearrangement, and the Co(II)‐HS state can go through the following transitions:

Co(II)-HS→Co(III)-HS→Co(III)-LS→Co(II)-LS

**Figure 5:**
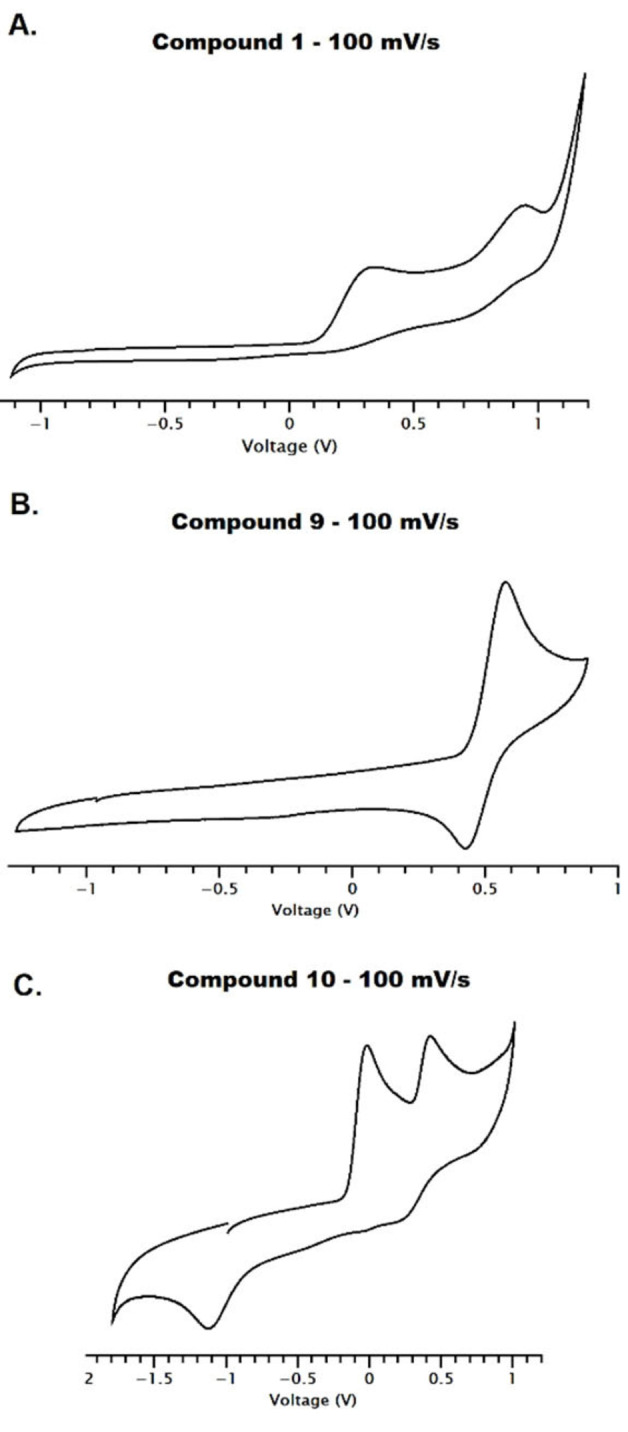
Cyclic voltammograms of compounds; **A**. complex **1**, **B**. complex **9** and **C**. complex **10** in dry DMF/0.1 M NBu_4_PF_6_; and at a scan rate=100 mV/s. Potentials are reported against ferrocene (Fc/Fc^+^=0.0 V).

Cyclic voltammetry measurements have been obtained for compounds **1**–**10** and assessed at scan rates between 100–800 mV/s (Figures S6–S15). At scan rates of 800 mV/s we observe only one oxidation for Co(II)→Co(III) (Epa_1_), however, at slower scan rates of 100 mV/s a second ligand‐based oxidation (Epa_2_) is observed (e.g. Figure 5A). Notably this second oxidation is not present for when X=chlorido (**9**) (Figure 5B) and suggests this is an ancillary ligand‐based oxidation. Complex **9** shows a *quasi‐*reversible oxidation/reduction, with Epa_1_ 0.58 V and Epc_1_ 0.43 V. In the case of the diisothiocyanato complexes (**1**–**8**), all are non‐reversible with weak reduction energies, which could not be fully identified (Table S4). There should be two reduction potential for the Co(III)‐Co(II) and ligand‐based reduction, however, 3–4 weak peaks are observed. Though these are not defined enough to calculate the energies, it is possible they are caused by the HS to LS electronic rearrangements noted by Harding and co‐workers. Complex **10** (X=H_2_O) has an enhanced reduction at −1.1 V, and due to the absence of this peak in the other voltammograms, this potential has been assigned to the aqua ligand (Figure 5C, Table S4).

**Antifungal activity**: *C. albicans* is the most prevalent human fungal pathogen and can cause life‐threatening invasive candidiasis. It is also predicted to contain a variety of anti‐oxidant enzymes.^[55]^ In the saliva, peroxidases can catalyze the oxidation of thiocyanate (−SCN) by hydrogen peroxide (H_2_O_2_) to hypothiocyanite (−OSCN) and hypothiocyanous acid (NCSOH),[^56^, ^57^] These compounds have been shown to inhibit the viability of *C. albicans*,^[58]^ and to the best of our knowledge, the role of the isothiocycanate has not yet been determined. Whereas, *A. fumigatus* is the primary cause of the devastating disease invasive aspergillosis, which is associated with mortality rates up to 90 %. Furthermore, azole resistance has emerged as a critical issue, and azole‐resistant *A. fumigatus* is to be listed as a priority fungal pathogen by the World Health Organization.

Previous reports have shown that Co(II) compounds exhibit moderate inhibition against fungal species, with slight increases in activity observed against both *A. flavus* and *C. albicans*, when compared to the activities of the free ligands, however, no selectivity was observed.^[23]^ Compounds **1**–**10** (at 32 μg ⋅ mL^−1^) were screened for their ability to inhibit the growth of the three common pathogenic species isolated from humans; *C. albicans*, *C. neoformans* and *A. fumigatus* (Table 2).^[59]^ Complexes with growth inhibition values >80 % were classed as active and complexes with growth inhibition values in the range 50–80 % were classed as partially active. All of our Co(II) complexes were completely inactive against *C. neoformans*, with no observed growth inhibition (0.0 %). The exact reasons underlying the striking inactivity of these compounds against *C. neoformans* is unknown but may be explained in part by the ability of this organism to form a polysaccharide capsule.^[60]^ Although the capsule of environmental isolates growing in laboratory media are much smaller (1–4 μm) and likely more porous than those grown in capsule‐inducing conditions, this capsule may be sufficient enough to inhibit the penetrance of the compounds tested and significantly affect their activity.[^61^, ^62^, ^63^] Notably, it has previously been shown that the capsule can protect *C. neoformans* against other antifungal drugs, such as the polyenes and glycolipid hydrolase inhibitors.[^64^, ^65^]

**Table 2:** Percentage growth inhibition at 32 μg ⋅ mL^−1^ against fungal species (*C. albicans, C. neoformans, A. fumigatus*) and bacterial species (*E. coli, K. pneumoniae, A. baumannii, P. aeruginosa, S. aureus*), IC_50_ values (μM) when screened against mammalian cell lines MIA PaCa‐2 and PNT2 (96 h) and CC_50_ values HEK293 (IC_50_=50 % inhibitory concentration in μM, CC_50_=50 % cytotoxic concentration in μg/mL, FCZ= Fluconazole, ITC=Itraconazole, CS=Colistin‐sulfate, VAN=Vancomycin, TAM=Tamoxifen, CIS=cisplatin).

	Fungal Species			Bacterial Species					Mammalian Cell Lines		
	Growth Inhibition [%]			Growth Inhibition [%]					IC_50_ [μM]		CC_50_ [μg ⋅ mL^−1^]
	*C. albicans*	*C. neoformans*	*A. fumigatus*	*E. coli*	*K. pneumoniae*	*A. baumannii*	*P. aeruginosa*	*S. aureus*	MIA PaCa‐2	PNT2	HEK 293
**1**	86.8	0.0	40.0	4.4	19.1	16.7	0.0	4.4	>100	>100	>32
**2**	78.6	0.0	32.0	5.2	15.6	11.7	0.0	5.2	>100	>100	>32
**3**	72.8	0.0	55.7	3.5	14.2	12.8	0.0	3.5	>100	>100	>32
**4**	81.0	0.0	73.6	0.0	19.2	12.5	0.0	0.0	>100	>100	>32
**5**	85.9	0.0	86.7	0.0	14.7	8.6	0.0	0.0	>100	>100	>32
**6**	73.5	0.0	73.3	0.0	16.2	12.8	0.0	0.0	>100	>100	>32
**7**	72.0	0.0	66.1	0.0	17.5	7.8	0.0	0.0	>100	>100	>32
**8**	63.5	0.0	54.3	2.1	17.3	11.7	0.0	2.1	>100	>100	>32
**9**	44.0	0.0	51.5	0.0	16.1	11.8	0.0	0.0	>100	>100	>32
**10**	26.0	0.0	45.7	3.1	11.2	6.9	0.0	3.1	>100	>100	>32
	MIC [μg ⋅ mL^−1^]			MIC [μg ⋅ mL^−1^]					IC_50_ [μM]		
FCZ	0.125	8.0	–	–	–	–	–	–	–	–	–
ITC	–	–	<2.5^[69]^	–	–	–	–	–	–	–	–
CS	–	–	–	0.125	0.25	0.25	0.25	–	–	–	–
VAN	–	–	–	–	–	–	–	1.0	–	–	–
TAM									–	–	9±2
CIS	–	–	–	–	–	–	–	–	3.6±0.3	8±1	–

All compounds (except **10**) show either partial activity (**2**, **3**, **6**–**9**) or are considered active (**1**, **4**, **5**) against *C. albicans*. The greatest inhibition was observed with the Co(II) compound containing an unsubstituted picolinamide ligand (**1**). However, changing the ancillary diisothiocyanato ligand in compound **1**, for dichlorido (**9**) or diaqua (**10**) significantly decreased the activity, by >1.9‐fold (**9**) or >3.3‐fold (**10**). Therefore, it can be concluded that the axial diisothiocyanato ligands are essential for the antifungal activity of the compounds against *C. albicans*. Fungal inhibition studies have been conducted on similar libraries of picolinamide ligands, however, MIC values of 100 μM and >200 μM were observed against both *C. albicans* and *C. neoformans*, respectively.^[66]^ This suggests the metal‐ligand combination is required for improved fungal inhibition at lower incubation concentrations.

**Figure 6:**
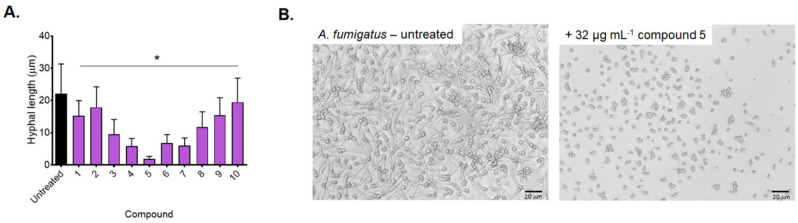
**A**. The average hyphal length of untreated A. fumigatus and following the treatment with compounds **1**–**10** after 24 h at 37 °C. **B**. A representative image depicting hyphal growth in the presence or absence of 32 μg ⋅ mL^−1^ compound **5** after 24 h. Hyphal length was measured using ImageJ and results are representative of 100 germinating fungal spores. Significance was determined by one‐way ANOVA with Dunnett's multiple comparisons. Scale bars represent 20 μm.

All compounds (except **2**) showed either show partial activity (50–80 %: **1**, **3**, **4**, **6**–**10**) or were considered active (>80 %: **5**) against *A. fumigatus*. Interestingly, the activity with compound **1** was lower against this fungal pathogen, and the relationship, **1**>**9**>**10** no longer applies, suggesting the diisothiocyanato ligands were not associated with high activity. Compound **5** (**L**, R=3’,5’‐diMe) displayed the greatest inhibition of 86.7 % and was also highly active against *C. albicans* (85.9 %), highlighting a broad spectrum of antifungal activity, which is desirable for drug development. The compounds were also screened at 16 μg ⋅ mL^−1^ and remain partially active (Figure S16). The growth of hyphae is associated with the invasive form of disease, whereby hyphae penetrate the vasculature and cause systemic aspergillosis. Treatment with compounds **4**–**7** had a demonstrable inhibitory effect on hyphal growth, although compound **5** demonstrated the most profound inhibition (Figure 6). The average length of hyphae treated with compound **5** was 91.4 % shorter than the hyphal length in the absence of the compound.

To confirm the selectivity of the compounds toward fungi, compounds **1**–**10** were additionally screened against bacterial strains (*E.coli, Klebsiella pneumoniae, Acinetobacter baumannii, Pseudomonas aeruginosa and S. aureus*)^[59]^ and mammalian cell lines: MIA PaCa‐2 (human pancreatic carcinoma), PNT2 (normal human prostate) and HEK293 (human embryonic kidney). Promising fungal drugs should not display cytotoxicity toward host cells. Here, we confirmed that all compounds demonstrate low toxicity toward bacterial species and are completely non‐toxic toward mammalian cells. This increases the potential of these compounds to be used as antifungal agents and warrants further drug design and development.

While all of the [(L)_2_Co(NCS)_2_] complexes were at least partially active, the three most active complexes (**1**, **4** and **5**) all contained the diisothiocyanato ligands in the *trans* arrangement. Although the differences in percentage growth inhibition were small, it is unknown if the positioning of the diisothiocyanate ligands was associated with the antifungal activity. However, thiocyanate is a known *trans*‐directing ligand and previous kinetic studies have been noted for *trans* and *cis* complexes with cobalt.[^67^, ^68^] In the *trans* configuration the isothiocyanates are released more readily, leading to greater intracellular concentrations, which could lead to the higher activities of these compounds.

## Conclusion

We have synthesized and characterized a range of bis(picolinamido)cobalt(II) complexes of the type, [(L)_2_Co(X)_2_]^0/2+^, where L=a neutral (*N,O*) picolinamide ligand and X=diisothiocyanato (NCS, **1**–**8**), dichlorido (Cl, **9**) or diaqua (H_2_O, **10**). SC‐XRD was determined for nine compounds, and PXRD was used to confirm the *cis* and *trans* ligand arrangements. Antifungal screening highlighted the high activity of these compounds toward fungi, with several compounds exhibiting growth inhibitions >80 % against both *C. albicans* and *A. fumigatus*. Notably the compounds have low activity against bacterial strains and no activity against mammalian cells, making them ideal candidates for further drug development. Compound **5** had the broadest range of antifungal activity and could inhibit the growth of *C. albicans* and *A. fumigatus* by 85.9 % and 86.7 %, respectively. Hyphae is associated with the invasive form of disease, and compound **5** was able to inhibit hyphal growth by 91.4 %, compared to the untreated control. Taken together, these bis(picolinamido)cobalt(II) complexes display antifungal activity toward clinically relevant fungal pathogens, low cytotoxicity toward the host, and represent a promising avenue for the development of novel antifungal drugs.

## Experimental Section

**General**: All ligands and complexes were synthesized under aerobic conditions. All ligands have previously been reported and ^1^H NMR used to confirm successful synthesis.[^42^, ^43^, ^44^, ^45^, ^46^, ^47^, ^48^, ^49^, ^50^] All chemicals were supplied by Sigma‐Aldrich Chemical Co., Acros Organics and Alfa Aesar. Deuterated NMR solvents were purchased from Sigma‐Aldrich Chemical Co. or Acros Organics.

**Instrumentation**: All NMR spectra were acquired using a Bruker Advance 300, 400 or 500 MHz spectrometer. Chemical shifts (δ) are expressed in parts per million (ppm) and reference to the solvent signal using an internal reference. Mass spectra were recorded at the University of Leeds Mass Spectrometry Service on a Bruker Daltonics MicroTOF instrument with electrospray ionization (ESI) and a photodiode array analyzer. Microanalyses were acquired either at the University of Leeds Microanalytical Service using a Carlo Erba 1108 Elemental Analyser or at the London Metropolitan University Elemental Analysis Service. Infrared spectra were acquired on a Perkin‐Elmer SpectrumOne FT‐IR spectrometer. Single crystal X‐ray diffraction data were collected using an Agilent (Rigaku) SuperNova X‐ray diffractometer fitted with an Atlas area detector using mirror monochromated Mo‐Ka (λ=0.71073 Å) radiation. X‐ray powder diffraction data were collected using a Bruker AXS D2Phaser diffractometer. Cyclic voltametric measurements were carried out using Autolab PGStat 30 potentiostat/galvanostat.

**Single Crystal X‐Ray Diffraction (SC‐XRD)**: The crystals were cooled to 120 K using an Oxford Cryosystem low temperature device.^[70]^ The full dataset was collected and the images processed using CrysAlisPro program.^[71]^ Structure solution by direct methods was achieved through the use of SHELXS86,^[72]^ SHELXL‐2014^[73]^ or SHELXT,^[74]^ and the structural model was refined by full matrix least squares on F^2^ using SHELX97^[75]^ interfaced through the program Olex2.^[76]^ Molecular graphics were plotted, editing of CIFs and construction of tables of bond lengths and angles were achieved using Olex2. Unless otherwise stated, hydrogen atoms were placed using idealized geometric positions (with free rotation for methyl groups), allowed to move in a “riding model” along with the atoms to which they were attached, and refined isotropically. Images were made using Mercury 4.0.^[77]^

**Powder X‐ray Diffraction (PXRD)**: Data collection was carried out at room temperature using Cu−Ka (λ=1.54184 Å) radiation. Diffraction patterns were recorded in step‐scan mode with a step size of 0.2° from 5° to 50° (5 s per step) using a 0.1 mm divergent slit. Experimental data was processed using Diffrac.Eva and Chekcell. Mercury was used to simulate the powder diffraction pattern from the single crystal structures.^[77]^

**Cyclic Voltammetry**: A single‐compartment or a conventional three‐electrode cell was used with a silver/silver chloride reference electrode (3 M NaCl, saturated AgCl), glassy carbon working electrode and Pt wire auxiliary electrode. Dimethylsulfoxide, DMSO, dimethylformamide, DMF or acetonitrile, MeCN was stored and used over molecular sieves (DMSO and DMF) or freshly distilled from CaH_2_ (MeCN). Tetrabutylammonium hexafluorophosphate [N(C_4_H_9_‐*n*)_4_][PF_6_] was used as the supporting electrolyte. Solutions containing ca. 10^−3^ M analyte (0.1 M electrolyte) were degassed by purging with argon, and spectra were collected with a constant flow of argon. All spectra were referenced to ferrocene/ferrocenium.

**Antifungal Screening (*C. albicans* and** ***C. neoformans*** **)**: Both screening and confirmation HITs were prepared as follows: Complexes were prepared in DMSO/water to a give a final concentration of 32 μg mL^−1^ in 384‐well NBS plates. The final DMSO concentration was at a maximum of 1.0 %. Fungi were cultured for 3 days on yeast extract‐peptone dextrose (YPD) agar at 30 °C. A yeast suspension of 1×10^6^ to 5×10^6^ CFU ⋅ mL^−1^ (as determined by OD_530_) was prepared from 5 colonies. The suspension was diluted and added to each well of the compound‐containing plates giving a final cell density of fungi suspension of 2.5×10^3^ CFU ⋅ mL^−1^ and a total volume of 50 μL. Fluconazole was used as a positive fungal inhibitor standard and provided in 4 concentrations, with 2 above and 2 below its MIC or CC_50_ value (plated into the first 8 wells of column 23 of the 384‐well NBS plates). All the plates were covered and incubated at 35 °C for 36 h without shaking and conducted in duplicate. Growth inhibition of *C. albicans* was determined measuring absorbance at 630 nm (OD_630_) and the growth inhibition of *C. neoformans* was determined measuring the difference in absorbance between 600 and 570 nm (OD_600–570_), after the addition of 0.001 % resazurin and incubation at 35 °C for an additional 2 h. The absorbance was measured using a Biotek Synergy HTX plate reader. The percentage of growth inhibition was calculated for each well, using the negative control (media only) and positive control (fungi without inhibitors) reference cells.

The significance of the inhibition values was determined by modified Z‐scores, calculated using the median and MAD of the samples (no controls) on the same plate. Samples with inhibition values ≥80 % and Z‐Score >2.5 for either replicate were classed as actives. Samples with inhibition values in the range 50–80 % and Z‐Score >2.5 for either replicate were classed as partial actives.

**Antifungal HIT Confirmation**: The MIC was determined as the lowest concentration at which the growth was fully inhibited, defined by an inhibition ≥80 % for *C. albicans* and an inhibition ≥70 % for *C. neoformans*. Due to a higher variance in growth and inhibition, a lower threshold was applied to the data for *C. neoformans*. In addition, the maximal percentage of growth inhibition is reported as D_Max_, indicating any compounds with marginal activity. Hits were classified by MIC <16 μg ⋅ mL^−1^ or 10 μM in either replicate.

**Antifungal Growth Inhibition (*A. fumigatus*)**: Complexes were prepared in DMSO to give a final concentration of 32 μg ⋅ mL^−1^ in a 96‐well plate. The final DMSO concentration was at a maximum of 1 %. *A. fumigatus* (a kind gift from Dr Alireza Abdolrasouli, Kings College University Hospital) was cultured on potato dextrose agar (PDA) for 5 days at 30 °C and ambient humidity. Spores were harvested using PBS containing 0.01 % (v/v) Tween‐80. Each well contained 2×10^4^ CFU ⋅ mL^−1^ (as determined via a haemocytometer count) in a total volume of 150 μL RPMI media. Plates were incubated for 24 h at 37 °C without shaking. Growth inhibition of *A. fumigatus* was determined measuring absorbance at 530 nm (OD_530_) following the addition of 0.001 % resazurin and incubation at 37 °C for an additional 2 h. Absorbance was measured using a Flexstation III multimode plate reader (Molecular Biosciences). The percentage of growth inhibition was calculated for each well, using the negative control (media only) and positive control (fungi without inhibitors) on the same plate. Images were obtained using an EVOS XL Core Imaging System (ThermoFisher Scientific) using a x40 objective. Hyphal growth was measured using ImageJ and is representative of 100 fungal cells. Significance was determined via one‐way ANOVA with Dunnett's multiple comparisons.

**Antibacterial Screening**: Both screening and confirmation HITs were prepared as follows: Complexes were prepared in DMSO to a give a final concentration of 32 μg ⋅ mL^−1^ in 384‐well non‐binding surface (NBS) plate. The final DMSO concentration was at a maximum of 1.0 %. All bacteria were cultured in Cation adjusted Mueller Hinton Broth (CAMHB) at 37 °C overnight. A sample of each culture was diluted 40‐fold in fresh broth and incubated at 37 °C for 1.5–3 h. The resultant mid‐log phase cultures were diluted (CFU ⋅ mL^−1^ measured by OD_600_), then added to each well of the compound containing plates, giving a cell density of 5×10^5^ CFU ⋅ mL^−1^ and a total volume of 50 μL. Colistin and vancomycin were used as positive bacterial inhibitor standards for Gram‐negative and Gram‐positive bacteria, respectively. Each standard was provided in 4 concentrations, with 2 above and 2 below its MIC or CC_50_ value (plated into the first 8 wells of column 23 of the 384‐well NBS plates). All plates were covered and incubated at 37 °C for 18 h without shaking and conducted in duplicate. Inhibition of bacterial growth was determined by measuring absorbance at 600 nm (OD_600_), using a Tecan M1000 Pro monochromator plate reader. The percentage of growth inhibition (%) was calculated for each well, using the negative control (media only) and positive control (bacteria without inhibitors) reference cells.

The significance of the inhibition values was determined by modified Z‐scores, calculated using the median and MAD (mean absolute deviation) of the samples (no controls) on the same plate. Samples with inhibition values ≥80 % and Z‐Score >2.5 for either replicate were classed as actives. Samples with inhibition values in the range 50–80 % and Z‐Score >2.5 for either replicate were classed as partial actives.

**Antibacterial HIT confirmation**: The percentage of growth inhibition was calculated as stated above and the MIC determined as the lowest concentration at which the growth was fully inhibited, defined by an inhibition ≥80 %. In addition, the maximal percentage of growth inhibition is reported as D_Max_, indicating any compounds with partial activity. HITs were classified by MIC <16 μg ⋅ mL^−1^ or 10 μM in either replicate.

### Mammalian Cytotoxicity Screening

*MIA PaCa‐2 and PNT2*: Cellular assays were conducted using human pancreatic carcinomas (MIA PaCa‐2) and human prostate cells (PNT2). All cell lines were routinely maintained as monolayer cultures in appropriate complete medium in either T‐25 or T‐75 flasks at 37 °C and 5 % CO_2_. All cell lines were culture in Dulbecco's modified eagle medium (DMEM) medium containing 10 % foetal calf serum supplemented with sodium pyruvate (1 mM) and L‐glutamine (2 mM). Prior to chemosensitivity studies, cell monolayers were passaged using Trypsin‐EDTA and diluted to a concentration of 1×10^4^ cells ⋅ mL^−1^. All assays were conducted using 96‐well plates, in which 100 μL of the cell suspension was added to each well and incubated for 24 h at 37 °C and 5 % CO_2_. After 24 h, 100 μL of the drug dilutions (100 mM stock in DMSO) in media were added to the plates (columns 3–12; column 1 contains media; column 2 contains 100 % cells), and then incubated for a further 96 h at 37 °C and 5 % CO_2_. After 96 h, 20 μL MTT (3‐(4,5‐dimethylthiazol‐2‐yl)‐2,5‐diphenyltetrazolium bromide, 5 mg ⋅ mL^−1^) was added to each well and incubated for 3 h at 37 °C and 5 % CO_2_. All solutions were then removed via pipette and 150 μL of DMSO added to each well to dissolve the purple formazan crystals. After mixing well, the absorbance of each well was measured at 540 nm using a ThermoFisher Multiskan FC spectrophotometer microplate reader. Results were plotted on a logarithmic scale, and the half maximal inhibitory concentration (IC_50_) determined from duplicate of triplicate repeats and reported as an IC_50_±Standard Deviation (SD).

*HEK293*: Cells were counted manually in a Neubauer hemocytometer and then plated in the 384‐well plates containing the compounds to give a density of 6×10^3^ cells ⋅ well^−1^ in a final volume of 50 μL. DMEM supplemented with 10 % FBS was used as growth media and the cells were incubated together with the compounds for 20 h at 37 °C in 5 % CO_2_. Tamoxifen was used as a positive cytotoxicity standard. The cytotoxic drug was provided at four concentrations, with two above and two below the CC_50_ value. All experiments were performed in duplicate. Cytotoxicity was measured by fluorescence with excitation at 560 nm and emission at 590 nm (F_560/590_), after addition of 5 μL of resazurin (25 μg ⋅ mL^−1^) and incubation for a further 3 h at 37 °C in 5 % CO_2_. The fluorescence intensity was measured using a Tecan M1000 Pro monochromator plate reader, using automatic gain calculation CC_50_ were calculated by curve fitting the inhibition values against log(concentration) using sigmoidal dose‐response function, with variable fitting values for bottom, top and slope. The maximal percentage of cytotoxicity is reported as D_Max_, indicating any compounds with partial cytotoxicity. The curve fitting was implemented using Pipeline Pilot's dose‐response component. Any value with >32 μg ⋅ mL^−1^ indicates a sample with no activity (low D_Max_ value) or samples with CC_50_ values above the maximum tested concentration. Cytotoxic samples were classified by CC_50_≤32 μg ⋅ mL^−1^ in either replicate.

**Synthesis and Characterization**: A functionalized picolinamide ligand (2 eq.) in ethanol (10 mL) was added to a solution of cobalt(II) nitrate hexahydrate (1 eq.) in distilled water (5 mL) and stirred at 50 °C for 15 min. Potassium thiocyanate (2 eq.) in distilled water (5 mL) was added dropwise and the mixture was stirred at 50 °C for 2 h to yield a dark pink solution. Slow evaporation of solvent from the reaction mixture yielded pure products.

**Complex 1**. Dark pink crystals. *Yield*: 0.175 g, 0.337 mmol, 73 %. ^*1*^ *H NMR: (d_6_‐DMSO, 300.13 MHz, 300.0 K)*: δ 10.54 (br. s, 2H, NH), 8.68 (br. s, 2H, pyridyl CH), 8.01 (br. s, 4H, pyridyl CH), 7.88 (br. s, 4H, aryl CH), 7.62 (br. s, 2H, pyridyl CH), 7.26 (br. s, 4H, aryl CH), 7.04 (br. s, 2H, aryl CH); *Analysis calculated for C_26_H_20_N_6_O_2_S_2_Co*: C 54.64, H 3.53, N 14.70 %, *Found*: C 54.45, H 3.50, N 15.10 %; *ES MS (+) (CH_3_CN): m/z* 513.07 [M‐NCS^+^]; *IR (cm^−1^)*: 3295 (br. w, NH), 3054 (w, CH), 2080 (s, CN), 1632 (m, CO), 1605 (m, CO), 1551 (m), 1341 (m), 1261 (m), 1019 (m), 903 (m), 747 (s), 706 (s), 508 (m).

**Complex 2**. Pink crystals. *Yield*: 0.228 g, 0.382 mmol, 91 %. ^*1*^ *H NMR: (d_6_‐DMSO, 300.13 MHz, 300.0 K)*: δ 10.21 (br. s, 2H, NH), 8.70 (br. s, 2H, pyridyl CH), 8.12 (br. s, 2H, pyridyl CH), 8.03 (br. s, 2H, pyridyl CH), 7.82 (br. s, 2H, aryl CH), 7.64 (br. s, 2H, pyridyl CH), 7.22 (br. s, 4H, aryl CH), 7.07 (br. s, 2H, aryl CH), 2.28 (br. s, 6H, CH_3_). *Analysis calculated for C_28_H_24_N_6_O_2_S_2_Co*: C 56.09, H 4.03, N 14.02 %. *Analysis found*: C 55.98, H 4.21, N 13.97 %. *ES MS (+) (CH_3_CN): m/z* 541.10 [M‐NCS^+^]. *IR (cm^−1^)*: 3225 (br. w, NH), 3066 (w, CH), 2872 (w, CH), 2069 (s, CN), 1632 (m, CO), 1589 (m, CO), 1523 (s), 1459 (m), 1304 (w), 997 (w), 917 (w), 747 (s), 692 (m), 595 (w).

**Complex 3**. Purple solid. *Yield*: 0.248 g, 0.395 mmol, 94 %. ^*1*^ *H NMR (d_6_‐DMSO, 500.23 MHz, 300.1 K)*: δ 10.20 (br. s, 2H, NH), 8.63 (br. s, 2H, pyridyl CH), 8.05 (br. s, 2H, pyridyl CH), 7.96 (br. s, 2H, aryl CH), 7.55 (br. s, 2H, pyridyl CH), 7.48 (br. s, 2H, pyridyl CH), 6.99 (br. s, 2H, aryl CH), 6.95 (br. s, 2H, aryl CH), 2.18 (br. s, 6H, methyl CH_3_), 2.09 (br. s, 6H, methyl CH_3_); *Analysis calculated for C_30_H_30_N_6_O_2_S_2_Co*: C 57.41, H 4.50, N 13.39 %, *Found*: C 57.50, H 4.64, N 13.24 %; *ES MS (+) (CH_3_CN: m/z* 569.13 [M‐NCS^+^]; *IR (cm^−1^)*: 3264 (br. w, NH), 3068 (w, CH), 2915 (w, CH), 2070 (s, CN), 1633 (m, CO), 1589 (m, CO), 1531 (s), 1471 (m), 1304 (w), 1264 (w), 1017 (w), 780 (m), 694 (m), 576 (w).

**Complex 4**. Pink crystals. *Yield*: 0.246 g, 0.410 mmol, 98 %. ^*1*^ *H NMR (d_6_‐DMSO, 300.13 MHz, 300.0 K)*: δ 10.46 (br. s, 2H, NH), 8.68 (br. s, 2H, pyridyl CH), 8.03 (br. s, 4H, pyridyl CH), 7.71 (br. s, 2H, aryl CH), 7.64 (br. s, 4H, aryl and pyridyl CH), 7.16 (br. s, 2H, aryl CH), 6.89 (br. s, 2H, aryl CH), 2.24 (br. s, 6H, CH_3_); *Analysis calculated for C_28_H_24_N_6_O_2_S_2_Co*: C 56.09, H 4.03, N 14.02 %, *Found*: C 55.97, H 4.02, N 13.95 %. *ES MS (+) (CH_3_CN): m/z* 541.10 [M‐NCS^+^]; *IR (cm^−1^)*: 3255 (br. w, NH), 3067 (w, CH), 2918 (w, CH), 2083 (s, CN), 1630 (s, CO), 1587 (m, CO), 1551 (s), 1438 (m), 1261 (w), 1020 (w), 781 (m), 682 (m), 645 (m).

**Complex 5**. Dark pink crystals. *Yield*: 0.233 g, 0.372 mmol, 89 %. ^*1*^ *H NMR (d_6_‐DMSO, 500.23 MHz, 300.1 K)*: δ 10.27 (br. s, 2H, NH), 8.60 (br. s, 2H, pyridyl CH), 7.96 (br. s, 4H, pyridyl CH), 7.45 (br. s, 6H, aryl and pyridyl CH), 6.67 (br. s, 2H, aryl CH), 2.12 (br. s, 12H, CH_3_); *Analysis calculated for C_30_H_28_N_6_O_2_S_2_Co*: C 57.41, H 4.50, N 13.39 %. *Found*: C 57.41, H 4.60, N 13.60 %; *ES MS (+) (CH_3_CN): m/z* 569.13 [M‐NCS^+^]; *IR (cm^−1^)*: 3263 (br. w, NH), 3099 (w, CH), 2912 (w, CH). 2098 (s, CN), 1632 (m, CO), 1557 (m, CO), 1433 (m), 1309 (w), 1020 (w), 824 (m), 746 (m), 678 (s).

**Complex 6**. Pink crystals. *Yield*: 0.174 g, 0.291 mmol, 69 %. ^*1*^ *H NMR (d_6_‐DMSO, 300.13 MHz, 300.0 K)*: δ 10.47 (br. s, 2H, NH), 8.68 (br. s, 2H, pyridyl CH), 8.03 (br. s, 4H, pyridyl CH), 7.75 (br. s, 4H, aryl CH), 7.61 (br. s, 2H, pyridyl CH), 7.08 (br. s, 4H, aryl CH), 2.20 (br. s, 6H, CH_3_); *Analysis calculated for C_28_H_26_N_6_O_3_S_2_Co ⋅ H_2_O*: C 54.45, H 4.24, N 13.79 %; *Found*: C 54.79, H 4.01, N 13.79 %; *ES MS (+) (CH_3_CN): m/z* 541.10 [M‐NCS^+^]; *IR (cm^−1^)*: 3261 (br. w, NH), 3085 (w, CH), 2081 (s, CN), 1630 (m, CO), 1586 (m, CO), 1529 (m), 1260 (w), 1020 (w), 808 (m), 751 (m), 687 (m), 596 (m), 509 (s).

**Complex 7**. Purple crystals. *Yield*: 0.127 g, 0.184 mmol, 44 %. ^*1*^ *H NMR (d_6_‐DMSO, 500.57 MHz, 299.3 K)*: δ 10.45 (br. s, 2H, NH), 8.66 (br. s, 2H, pyridyl CH), 8.07 (br. s, 2H, pyridyl CH), 8.01 (br. s, 2H, pyridyl CH), 7.60 (br. s, 2H, pyridyl H), 7.19 (br. s, 4H, aryl CH), 6.21 (br. s, 2H, aryl CH), 3.60 (br. s, 6H, OCH_3_); Analysis calculated *for C_30_H_28_N_6_O_6_S_2_Co*: C 52.10, H 4.08, N 12.15 %; *Found*: C 51.97, H 4.09, N 12.01 %; *ES MS (+) (CH_3_CN): m/z* 633.11 [M‐NCS^+^]; *IR (cm^−1^)*: 3207 (br. w, NH), 3084 (w, CH), 2963 (w, CH), 2072 (s, CN), 1612 (m, CO), 1555 (m, CO), 1318 (m), 1158 (s), 1065 (m), 832 (m), 752 (m), 675 (m), 640 (m).

**Complex 8**. Pink crystals. *Yield*: 0.146 g, 0.231 mmol, 55 %. ^*1*^ *H NMR (d_6_‐DMSO, 500.57 MHz, 299.3 K)*: δ 10.47 (br. s, 2H, NH), 8.68 (br. s, 2H, pyridyl CH), 8.09 (br. s, 2H, pyridyl CH), 8.01 (br. s, 2H, pyridyl CH), 7.78 (br. s, 4H, aryl CH), 7.61 (br. s, 2H, pyridyl CH), 6.88 (br. s, 4H, aryl CH), 3.49 (br. s, 6H, OCH_3_); *Analysis calculated for C_28_H_24_N_6_O_4_S_2_Co*: C 53.25, H 3.83, N 13.31 %. *Analysis found*: C 53.33, H 3.70, N 13.24 %; *ES MS (+) (CH_3_CN): m/z* 573.09 [M‐NCS^+^]; *IR (cm^−1^)*: 3300 (br. w, NH), 3092 (w, CH), 2837 (w, CH), 2081 (s, CN), 1626 (m, CO), 1585 (m, CO), 1537 (m), 1508 (m), 1186 (m), 1020 (m), 827 (m), 751 (m), 550 (m), 524 (m).

**Complex 9**. A functionalized picolinamide ligand (2 eq.) in ethanol (10 mL) was added to a solution of cobalt(II) chloride hexahydrate (1 eq.) in ethanol (10 mL) and stirred at 50 °C for 2 h to yield an orange suspension. The solid was filtered, washed with ethanol and dried to yield pure product. Orange crystals. *Yield*: 0.212 g, 0.403 mmol, 96 %. ^*1*^ *H NMR: (d_6_‐DMSO, 300.13 MHz, 300.0 K)* δ 10.59 (br. s, 2H, NH), 8.74 (br. s, 2H, pyridyl CH), 8.10 (br. s, 4H, pyridyl CH), 7.91 (br. s, 4H, aryl CH), 7.68 (br. s, 2H, pyridyl CH), 7.33 (br.s, 4H, aryl CH), 7.10 (br.s, 2H, aryl CH). *Analysis calculated for C_24_H_20_Cl_2_N_4_O_2_Co*: C 54.77, H 3.83, N 10.65 %. *Analysis found*: C 54.86, H 3.65, N 10.62 %. *ES MS (+) (CH_3_CN): m/z* 490.06 [M–Cl^+^]. *IR (cm^−1^)*: 3248 (w, NH), 3092 (w, ArCH), 1630 (m, CO), 1609 (m, CO), 1586 (m), 1558 (m), 1502 (m), 1447 (m), 1347 (m), 1264 (w), 1022 (w), 904 (w), 758 (s), 681 (s), 515 (m).

**Complex 10**. A functionalized picolinamide ligand (2 eq.) in ethanol (10 mL) was added to a solution of cobalt(II) chloride hexahydrate (1 eq.) in distilled water (5 mL) and stirred at 50 °C for 15 min. Potassium iodide (2 eq.) in distilled water (5 mL) was added dropwise and the mixture was stirred at 50 °C for 2 h to yield an orange solution. Slow evaporation of solvent from the reaction mixture yielded pure product. Orange crystals. *Yield*: 0.304 g, 0.408 mmol, 97 %. ^*1*^ *H NMR (d_6_‐DMSO, 300.13 MHz, 300.0 K)*: δ 10.54 (2H, NH), 8.68 (br. d, 2H, pyridyl CH), 8.11 (br. s, 4H, pyridyl CH), 7.86 (br. s, 4H, aryl CH), 7.62 (br.s, 2H, pyridyl CH), 7.25 (br. s, 4H, H pyridyl CH), 7.06 (br. s, 2H, pyridyl CH); *Analysis calculated for C_24_H_24_I_2_N_4_O_2_Co* ⋅ *3H_2_O*: C 36.07, H 3.78, N 7.01 %; *Found*: C 35.85, H 3.18, N 6.66 % *ES MS (+) (CH_3_CN:) m/z* 582.00 [M‐(2H_2_O+I)]^+^; *IR (cm^−1^)*: 3315 (br. m, OH), 3254 (m, NH), 3084 (w, ArCH), 1626 (m, CO), 1602 (m, CO), 1582 (m), 1547 (m), 1447 (m), 1344 (w), 1262 (w), 1054 (w), 899 (w), 765 (s), 688 (s), 585 (s).

## Conflict of interest

The authors declare no conflict of interest.

## Supporting information

As a service to our authors and readers, this journal provides supporting information supplied by the authors. Such materials are peer reviewed and may be re‐organized for online delivery, but are not copy‐edited or typeset. Technical support issues arising from supporting information (other than missing files) should be addressed to the authors.

Supporting Information
