# Supplementary material for: Bis(N‐picolinamido)cobalt(II) Complexes Display Antifungal Activity toward Candida albicans and Aspergillus fumigatus
Source: ChemMedChem. 2021 Jul 29;16(20):3210–21. doi: 10.1002/cmdc.202100159 (PMC8597028; doi:10.1002/cmdc.202100159)
Supplement: Supplementary file 1 — Supporting Information [file CMDC-16-3210-s001.pdf]

ChemMedChem

Supporting Information

Bis(*N*-picolinamido)cobalt(II) Complexes Display Antifungal Activity toward *Candida albicans* and *Aspergillus fumigatus*

Laura H. D. Ghandhi, Stefan Bidula, Christopher M. Pask, Rianne M. Lord,* and Patrick C. McGowan*

Supporting Information

Single Crystal X-ray Diffraction (SC-XRD)

Table S 1 *X-ray crystallographic data for complexes **1, 2, 4** and **5** s.u.s in parenthesis (λ = 0.71073 Mo-Kα)*

Complex	1	2	4	5
CCDC	2065377	2065381	2065375	2065376
Empirical formula	$C_{26}H_{20}CoN_6O_2S_2$	$C_{28}H_{24}CoN_6O_2S_2$	$C_{28}H_{24}CoN_6O_2S_2$	$C_{30}H_{28}CoN_6O_2S_2$
Formula weight	571.553	599.607	599.607	627.661
Temperature/K	120.00(10)	119.99(17)	120.00(13)	120.01(10)
Crystal system	triclinic	monoclinic	monoclinic	triclinic
Space group	*P*-1	*P*2₁/c	*P*2₁/c	*P*-1
a/Å	8.1387(6)	10.4338(14)	8.0460(9)	7.8650(8)
b/Å	8.7460(5)	13.6460(14)	21.181(2)	8.4741(8)
c/Å	9.8413(7)	19.816(3)	8.9024(11)	12.8131(13)
α/°	85.840(6)	90	90	105.541(9)
β/°	75.883(6)	103.722(16)	113.347(14)	91.317(8)
γ/°	66.742(7)	90	90	116.677(10)
Volume/Å3	623.93(8)	2740.9(7)	1392.9(3)	724.92(15)
Z	1	4	2	1
ρcalcg/cm3	1.521	1.453	1.430	1.438
μ/mm 1	0.893	0.816	0.803	0.775
F(000)	293.7	1238.9	619.4	325.7
Crystal size/mm3	0.19 × 0.14 × 0.11	0.16 × 0.12 × 0.08	0.13 × 0.11 × 0.04	0.2 × 0.14 × 0.09
Radiation	Mo Kα (λ = 0.71073)	MoKα (λ = 0.71073)	MoKα (λ = 0.71073)	MoKα (λ = 0.71073)
2Θ range for data collection/°	6.54 to 56.56	5.98 to 62.46	5.84 to 62.5	6.26 to 62.3
Index ranges	-10 ≤ h ≤ 10, -11 ≤ k ≤ 11, -13 ≤ l ≤ 13	-15 ≤ h ≤ 10, -19 ≤ k ≤ 19, -26 ≤ l ≤ 28	-9 ≤ h ≤ 11, -28 ≤ k ≤ 29, -12 ≤ l ≤ 7	-11 ≤ h ≤ 10, -11 ≤ k ≤ 12, -17 ≤ l ≤ 11
Reflections collected	12343	21138	9421	5365
Independent reflections	3094 [R_{int} = 0.0458, R_{sigma} = 0.0405]	7795 [R_{int} = 0.0911, R_{sigma} = 0.1232]	3988 [R_{int} = 0.0477, R_{sigma} = 0.0761]	3534 [R_{int} = 0.0333, R_{sigma} = 0.0633]
Data/restraints /parameters	3094/0/169	7795/0/354	3988/0/179	3534/0/189
Goodness-of-fit on F2	1.047	1.061	1.059	1.046
Final R indexes [I>=2σ (I)]	R_1 = 0.0354, wR_2 = 0.0772	R_1 = 0.0840, wR_2 = 0.1937	R_1 = 0.0545, wR_2 = 0.1139	R_1 = 0.0636, wR_2 = 0.1558
Final R indexes [all data]	R_1 = 0.0429, wR_2 = 0.0824	R_1 = 0.1316, wR_2 = 0.2288	R_1 = 0.0929, wR_2 = 0.1341	R_1 = 0.0807, wR_2 = 0.1720
Largest diff. peak/hole / e Å-3	0.38/-0.37	2.28/-1.35	0.71/-0.73	1.33/-0.72

Table S 2 *X-ray crystallographic data for complexes **6-10**, s.u.s in parenthesis (λ = 0.71073 Mo-Kα)*

Complex	6	7	8	9	10
**CCDC**	**2065378**	**2065383**	**2065379**	**2065380**	**2065382**
Empirical formula	$C_{28}H_{24}CoN_6O_2S_2$	$C_{30}H_{28}CoN_6O_6S_2$	$C_{28}H_{24}CoN_6O_4S_2$	$C_{24}H_{20}Cl_2CoN_4O_2$	$C_{24}H_{24}CoI_2N_4O_4$
Formula weight	599.607	691.630	631.606	526.290	745.225
Temperature/K	120.0(2)	119.99(14)	120.2(6)	120.01(12)	120.01(10)
Crystal system	monoclinic	triclinic	monoclinic	monoclinic	monoclinic
Space group	*C*c	*P*-1	*C*c	$P2_1$/c	$P2_1$/c
a/Å	8.2408(7)	14.4852(5)	8.51272(16)	10.3023(12)	8.2666(2)
b/Å	20.8725(16)	15.8534(5)	20.4558(4)	12.9556(8)	11.2691(3)
c/Å	16.2881(13)	27.0438(8)	16.2386(3)	9.3713(10)	14.7572(5)
α/°	90	90.006(2)	90	90	90
β/°	98.690(8)	90.054(3)	97.6694(17)	115.732(13)	105.541(3)
γ/°	90	104.182(3)	90	90	90
Volume/Å3	2769.5(4)	6021.1(3)	2802.40(9)	1126.8(2)	1324.49(7)
Z	4	8	2	2	2
ρcalcg/cm3	1.438	1.526	1.497	1.551	1.869
μ/mm 1	0.808	0.764	6.585	1.029	3.015
F(000)	1238.9	2856.0	1297.6	539.5	721.4
Crystal size/mm3	0.35 × 0.07 × 0.04	0.31 × 0.14 × 0.10	0.24 × 0.07 × 0.02	0.1336 × 0.1119 × 0.0886	0.24 × 0.16 × 0.07
Radiation	MoKα (λ = 0.71073)	Mo Kα (λ = 0.71073)	CuKα (λ = 1.54184)	Mo Kα (λ = 0.71073)	MoKα (λ = 0.71073)
2Θ range for data collection/°	6.24 to 62.70	5.59 to 62.69	8.64 to 147.44	5.76 to 62.42	6.26 to 62.4
Index ranges	-8 ≤ h ≤ 11, -30 ≤ k ≤ 20, -23 ≤ l ≤ 18	-20 ≤ h ≤ 20, -22 ≤ k ≤ 23, -38 ≤ l ≤ 33	-10 ≤ h ≤ 9, -24 ≤ k ≤ 17, -18 ≤ l ≤ 19	-15 ≤ h ≤ 11, -12 ≤ k ≤ 18, -9 ≤ l ≤ 13	-8 ≤ h ≤ 11, -15 ≤ k ≤ 16, -19 ≤ l ≤ 21
Reflections collected	10913	67052	5343	6848	11519
Independent reflections	5736 [R_{int} = 0.0497, R_{sigma} = 0.0818]	31516 [R_{in} = 0.0525, R_{sigma} = 0.0879]	3493 [R_{in} = 0.0282, R_{sigma} = 0.0411]	3193 [R_{in} = 0.0371, R_{sigma} = 0.0636]	3880 [R_{in} = 0.0552, R_{sigma} = 0.0617]
Data/restraints/parameters	5736/2/354	31516/0/1638	3493/2/372	3193/0/151	3880/0/161
Goodness-of-fit on F2	1.037	1.006	1.060	1.044	1.037
Final R indexes [I>=2σ (I)]	R_1 = 0.0513, wR_2 = 0.0797	R_1 = 0.0550, wR_2 = 0.1025	R_1 = 0.0298, wR_2 = 0.0759	R_1 = 0.0500, wR_2 = 0.0995	R_1 = 0.0407, wR_2 = 0.0810
Final R indexes [all data]	R_1 = 0.0655, wR_2 = 0.0851	R_1 = 0.0883, wR_2 = 0.1179	R_1 = 0.0312, wR_2 = 0.0770	R_1 = 0.0692, wR_2 = 0.1078	R_1 = 0.0540, wR_2 = 0.0942
Largest diff. peak/hole / e Å-3	0.67/-0.45	0.74/-0.69	0.20/-0.24	0.92/-0.60	1.81/-1.33
Flack parameter	-0.006(17)	--	-0.022(2)	--	--

***Table S 3** Selected bond angles (°) for complexes **1, 2** and **4-10**, s.u.s in parenthesis*

	Angle (°)					
	**N(1)-Co(1)-N(1')**	**O(1)-Co(1)-O(1')**	**N(1)-Co(1)-O(1)/ N(1)-Co(1)-O(1')**	**N(1)-Co(1)-X(1/1')**	**O(1)-Co(1)-X(1/1')**	**X(1)-Co(1)-X(1')**
**1**[a]	180.0	180.0[a]	77.29(5)/ 102.71(5)	89.32(6)/ 90.68(6)	91.39(6)/ 88.61(6)	180.0
**2**	158.17(14)	85.89(12)	87.56(12)/ 76.51(12)/ 75.90(12)/ 88.02(12)	94.36(14)/ 99.52(14)/ 99.31(14)/ 96.22(14)	89.40(14)/ 169.88(14)/ 171.59(14)/ 90.97(14)	94.88(16)
**4**[a]	180.0	180.0	76.86(8)/ 103.14(8)	88.68(9)/ 91.32(9)	89.59(8)/ 90.41(8)	180.0
**5**[a]	180.0	180.0	103.19(8)/ 76.81(8)	86.37(10)/ 93.63(10)	92.24(10)/ 87.76(10)	180.0
**6**	165.87(11)	88.41(10)	76.68(10)/ 93.47(11)/ 75.94(11)/ 93.46(10)	91.07(12)/ 95.66(12)/ 97.56(13)/ 91.07(12))	171.61(13)/ 97.56(13)/ 87.46(11)/ 170.41(11)	99.13(13)
**7; Co1**	166.01(19)	75.02(14)	76.51(14)/ 96.04(15)/ 90.62(15)/ 75.03(16)	93.49(17)/ 98.04(17)/ 91.40(17)/ 94.00(17)	165.24(17)/ 95.62(17)/ 89.63(17)/ 160.79(16)	101.6(2)
**Co2**	169.54(19)	74.39(14)	94.61(15)/ 75.33(15)/ 75.76(14)/ 97.80(15)	95.98(19)/ 92.72(17)/ 92.84(18)/ 91.16(17)	164.21(17)/ 96.98(19)/ 89.46(18)/ 158.85(17)	101.7(2)
**Co3**	169.38(19)	74.42(15)	76.21(14)/ 98.56(15)/ 93.86(15)/ 74.80(16)	87.85(17)/ 95.78(17)/ 92.74(17)/ 96.18(18)	88.70(17)/ 159.79(17)/ 163.97(17)/ 96.21(18)	102.6(2)
**Co4**	165.74(18)	75.42(14)	76.37(14)/ 96.39(15)/ 90.13(15)/ 75.44(16)	94.21(17)/ 98.13(18)/ 88.71(17)/ 95.66(17)	165.68(17)/ 95.27(18)/ 88.76(18)/ 161.63(17)	102.0(2)
**8**	165.92(10)	88.38(9)	76.09(9)/ 93.43(9)/ 93.06(9)	89.96(11)/ 94.63(10)/ 98.22(11)	85.49(10)/ 172.09(11)/ 169.54(10)	99.50(11)
**9**	180.0	180.0	77.42(7)/ 102.58(7)	90.35(6)/ 89.65(6)	90.13(5)/ 89.87(5)	180.0
**10**[a]	180.0	180.0	77.87(10)/ 102.13(10)	86.29(11)/ 93.71(11)	91.72(10)/ 91.72(10)	180.0

[a] = symmetry generated

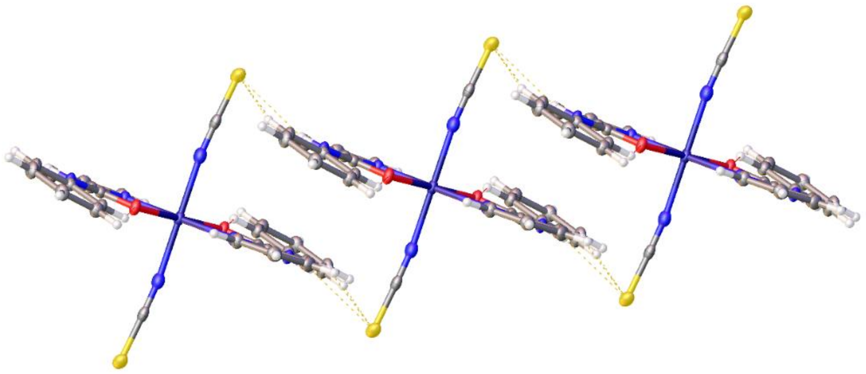

Figure S 1 *Intermolecular hydrogen bonding and π-π stacking interactions in complex* ***1.***

NMR Spectroscopy

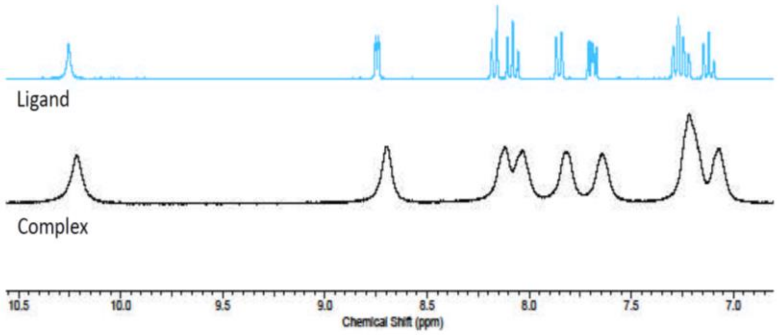

Figure S 2 *^{1}H NMR spectra of complex* ***2*** *and the corresponding ligand* ***L2*** *(d_6-DMSO, 500.23 MHz, 300.1 K).*

UV-vis Spectroscopy

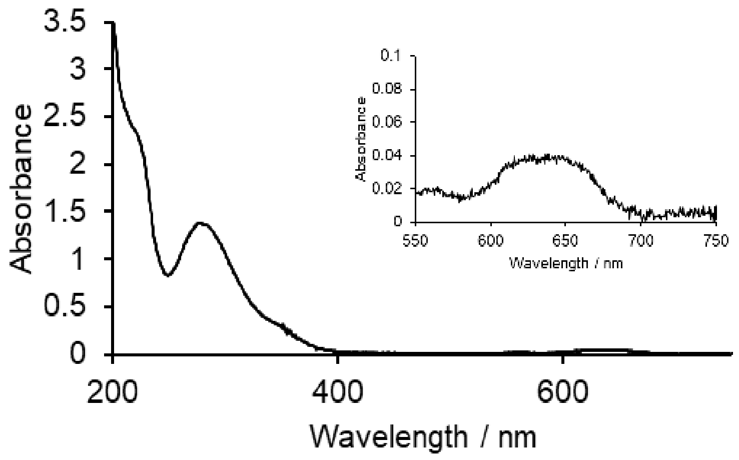

Figure S 3 *UV-vis spectrum for complex* ***1.*** *Intense ligand-based absorbance (π-π*) at ~200 nm with two less intense ~ 280 nm and a weak d-d transition at around 640 nm.*

Powder X-ray Diffraction (PXRD)

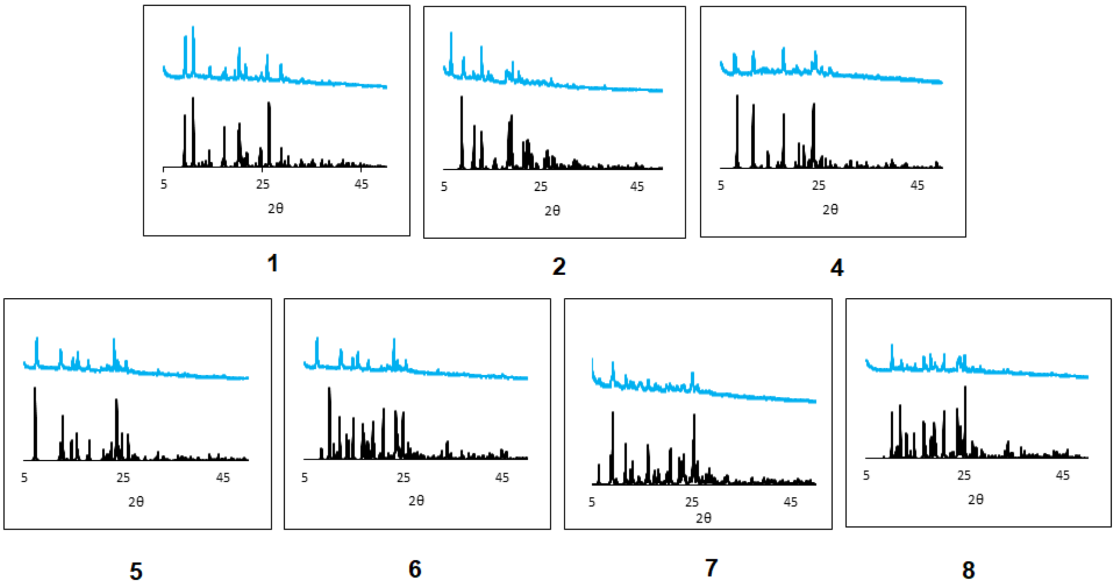

Figure S 4 *Powder X-ray Diffraction (PXRD) patterns for complexes* ***1***, ***2*** *and* ***4-8***, *black = simulated and blue = experimental.*

Magnetic Measurements

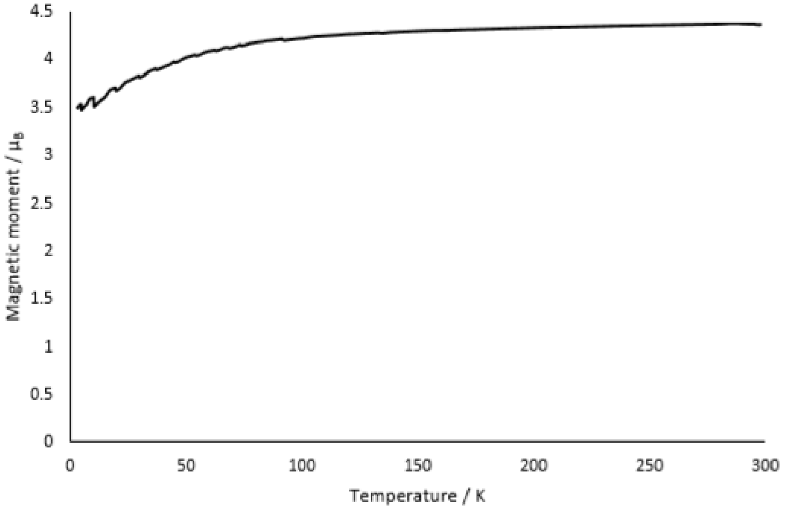

Figure S 5 *Variable temperature magnetic moments measurements of complex* ***2.***

Cyclic Voltammetry

Table S 4 *Cyclic voltametric analysis of compounds* ***1-10*** *in dry DMF/ 0.1 M NBu_4PF_6; at a scan rate = 100 mV/s. Potentials are reported against ferrocene (Fc/Fc^+ = 0.0 V)*

	Epa$_1$	Epa$_2$	Epc$_2$	Epc$_1$	ΔEp$_1$	ΔEp$_2$
**1**	0.33	0.95	w	w	nd	nd
**2**	0.38	1.02	w	w	nd	nd
**3**	0.29	0.98	w	w	nd	nd
**4**	0.31	0.95	w	w	nd	nd
**5**	0.32	0.97	w	w	nd	nd
**6**	0.34	nv	w	w	nd	nd
**7**	0.25	1.03	w	w	nd	nd
**8**	0.24	0.84	w	w	nd	nd
**9**	0.58	nv	nv	0.43	0.14	nd
**10**	-0.02	0.42	0.25	-1.12	1.11	0.18

nv = not visible
nd = not determined
w = weak

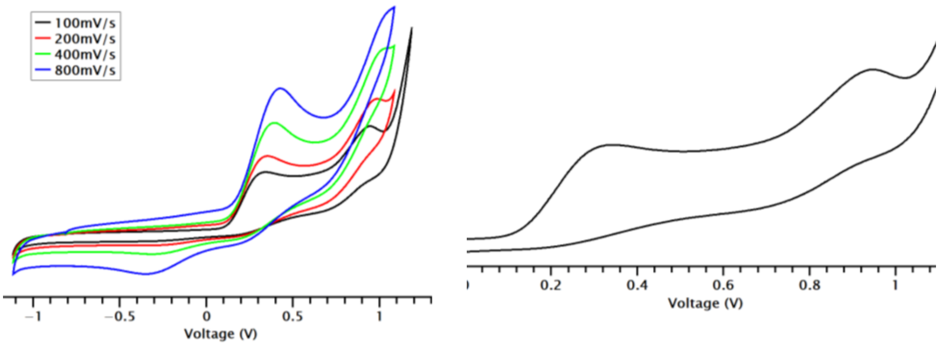

scan rate = 100 - 800 mV/s | scan rate = 100 mV/s (cut)

Figure S 6 *Cyclic voltammograms of compound* ***1*** *in dry DMF/ 0.1 M NBu_4PF_6. Potentials are reported against ferrocene (Fc/Fc^+ = 0.0 V).*

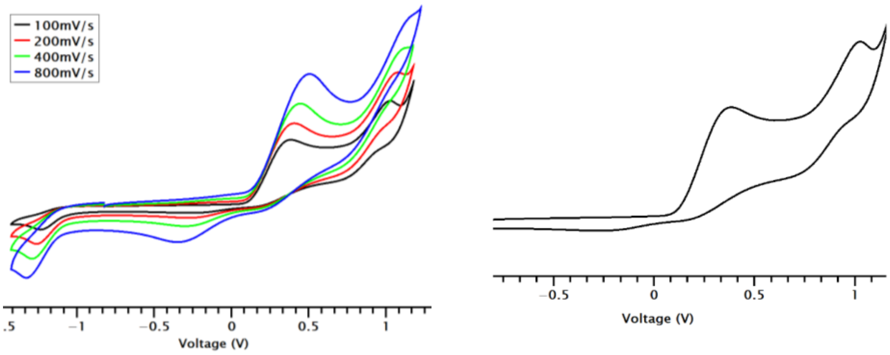

scan rate = 100 - 800 mV/s | scan rate = 100 mV/s (cut)

Figure S 7 *Cyclic voltammograms of compound* ***2*** *in dry DMF/ 0.1 M NBu_4PF_6. Potentials are reported against ferrocene (Fc/Fc^+ = 0.0 V).*

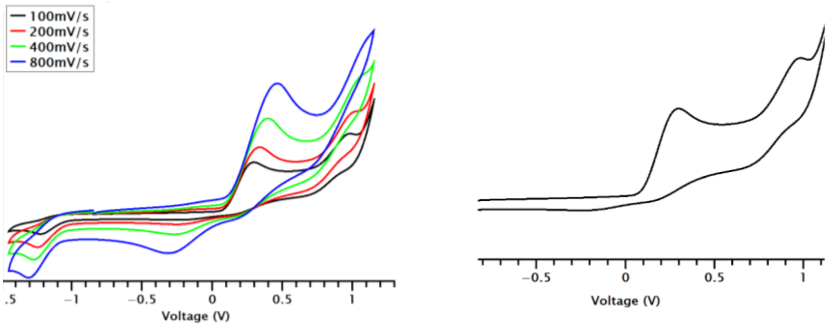

scan rate = 100 - 800 mV/s scan rate = 100 mV/s (cut)

Figure S 8 *Cyclic voltammograms of compound* ***3*** *in dry DMF/ 0.1 M NBu_4PF_6. Potentials are reported against ferrocene (Fc/Fc^+ = 0.0 V).*

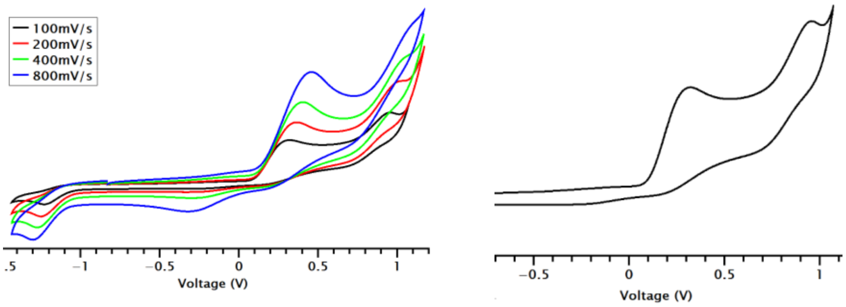

scan rate = 100 - 800 mV/s scan rate = 100 mV/s (cut)

Figure S 9 *Cyclic voltammograms of compound* ***4*** *in dry DMF/ 0.1 M NBu_4PF_6. Potentials are reported against ferrocene (Fc/Fc^+ = 0.0 V).*

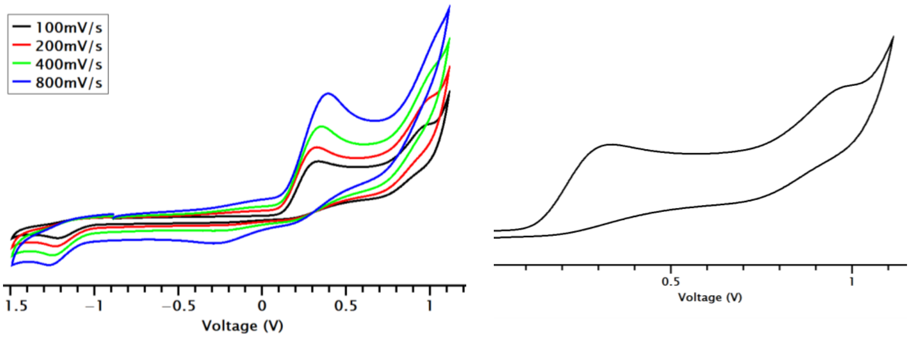

scan rate = 100 - 800 mV/s scan rate = 100 mV/s (cut)

Figure S 10 *Cyclic voltammograms of compound* ***5*** *in dry DMF/ 0.1 M NBu_4PF_6. Potentials are reported against ferrocene (Fc/Fc^+ = 0.0 V).*

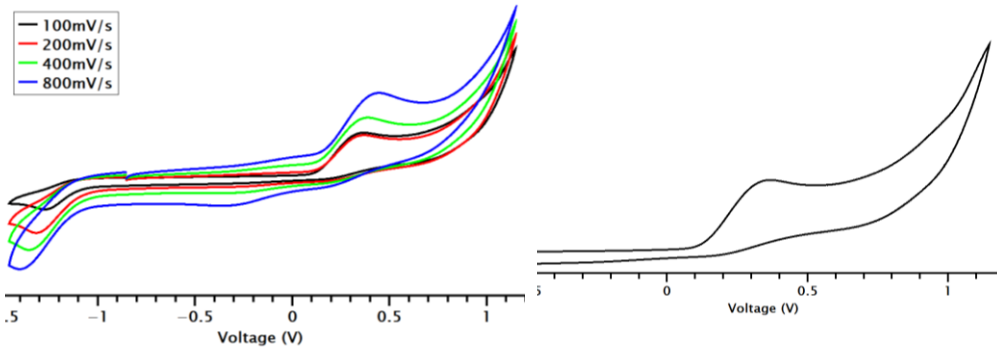

scan rate = 100 - 800 mV/s

scan rate = 100 mV/s (cut)

Figure S 11 *Cyclic voltammograms of compound **6** in dry DMF/ 0.1 M NBu_4PF_6. Potentials are reported against ferrocene (Fc/Fc^+ = 0.0 V).*

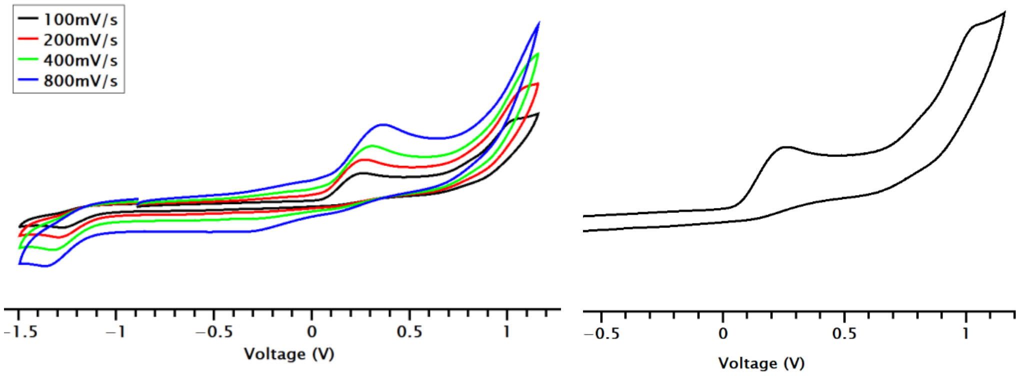

scan rate = 100 - 800 mV/s

scan rate = 100 mV/s (cut)

Figure S 12 *Cyclic voltammograms of compound **7** in dry DMF/ 0.1 M NBu_4PF_6. Potentials are reported against ferrocene (Fc/Fc^+ = 0.0 V).*

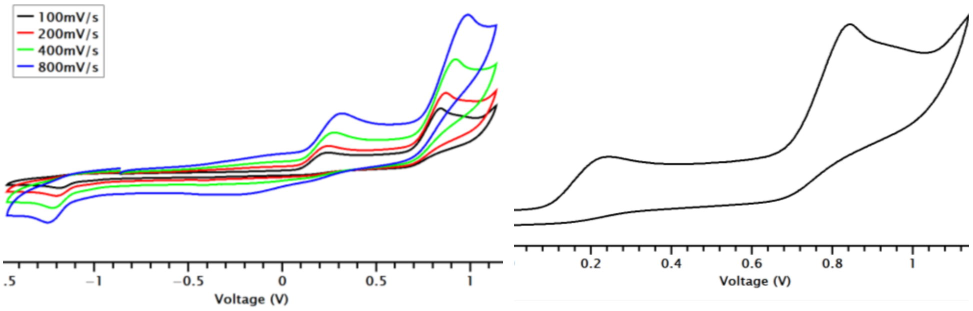

scan rate = 100 - 800 mV/s

scan rate = 100 mV/s (cut)

Figure S 13 *Cyclic voltammograms of compound **8** in dry DMF/ 0.1 M NBu_4PF_6. Potentials are reported against ferrocene (Fc/Fc^+ = 0.0 V).*

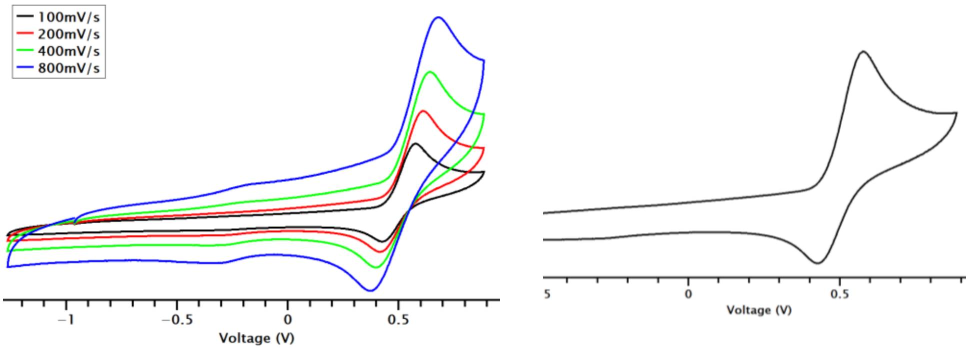

scan rate = 100 - 800 mV/s | scan rate = 100 mV/s (cut)

Figure S 14 *Cyclic voltammograms of compound **9** in dry DMF/ 0.1 M NBu_4PF_6. Potentials are reported against ferrocene (Fc/Fc^+ = 0.0 V).*

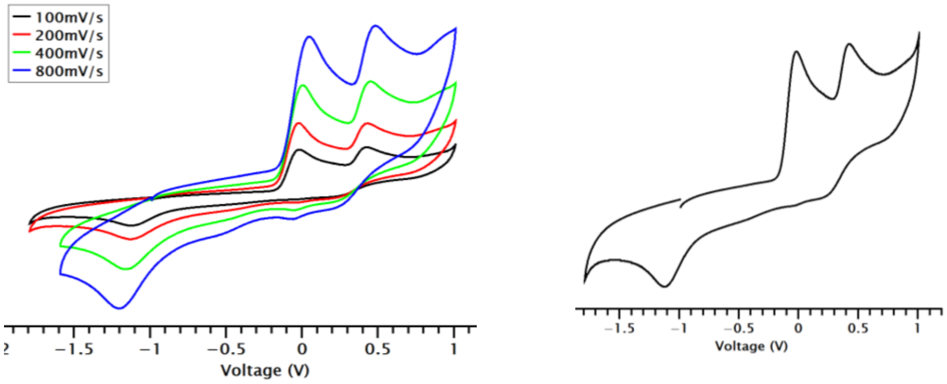

scan rate = 100 - 800 mV/s | scan rate = 100 mV/s (full)

Figure S 15 *Cyclic voltammograms of compound **10** in dry DMF/ 0.1 M NBu_4PF_6. Potentials are reported against ferrocene (Fc/Fc^+ = 0.0 V).*

Anti-bacterial Activity

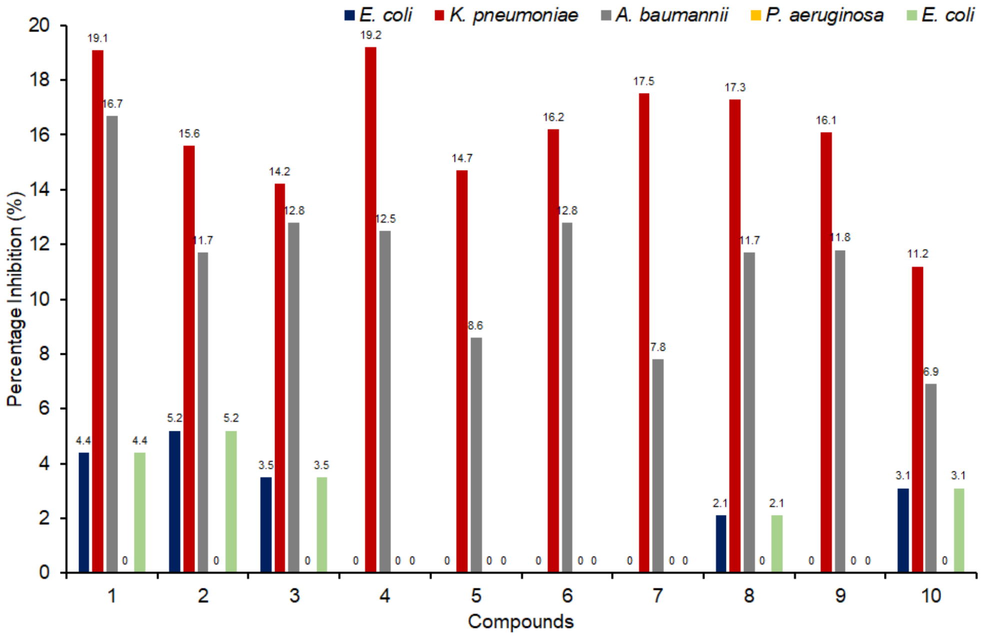

Figure S 16 *The growth inhibition of E. coli, K. pneumoniae, A. baumannii, P. aeuginosa and E.coli in the presence of 32 μg·mL^{-1} of compounds **1-10** after 24 h incubation at 37 °C. Compounds are all inactive, with inhibition values <40%.*

Anti-fungal Activity

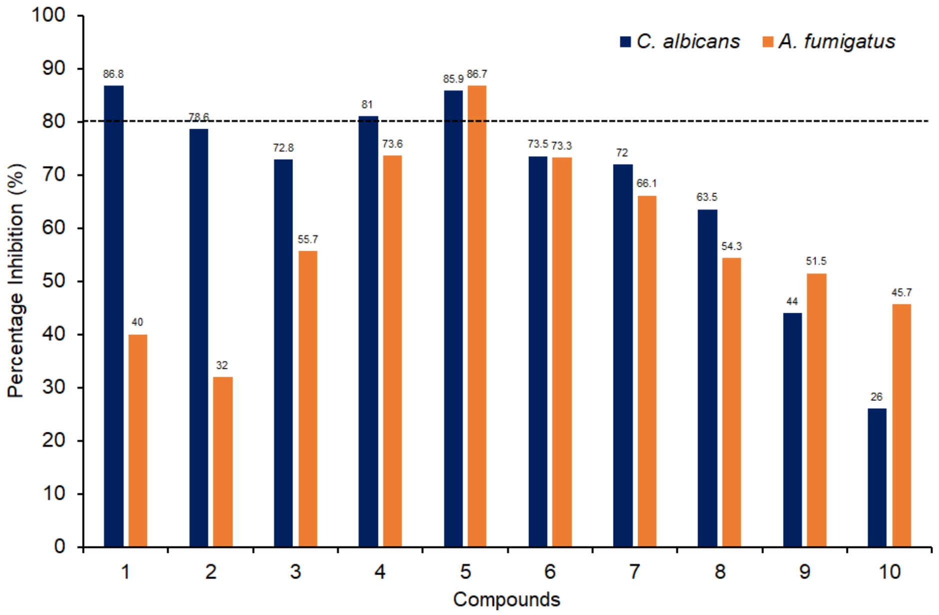

Figure S 17 *The growth inhibition of C. albicans and A.fumigatus in the presence of 32 μg·mL^{-1} of compounds* ***1-10*** *after 24 h incubation at 37 °C. Compounds with activity above 80% (dashed line) were considered active.*

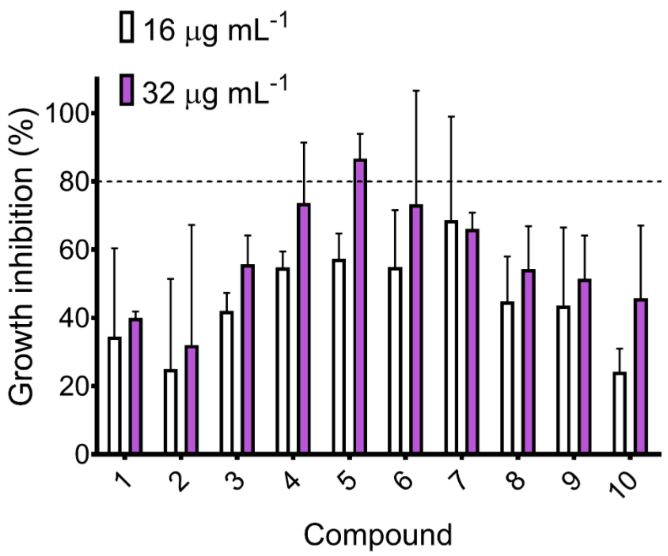

Figure S 18 *The growth inhibition of A. fumigatus in the presence or absence of 16 or 32 μg·mL^{-1} of compounds* ***1-10*** *after 24 h incubation at 37 °C. Compounds with activity above 80% (dashed line) were considered active.*

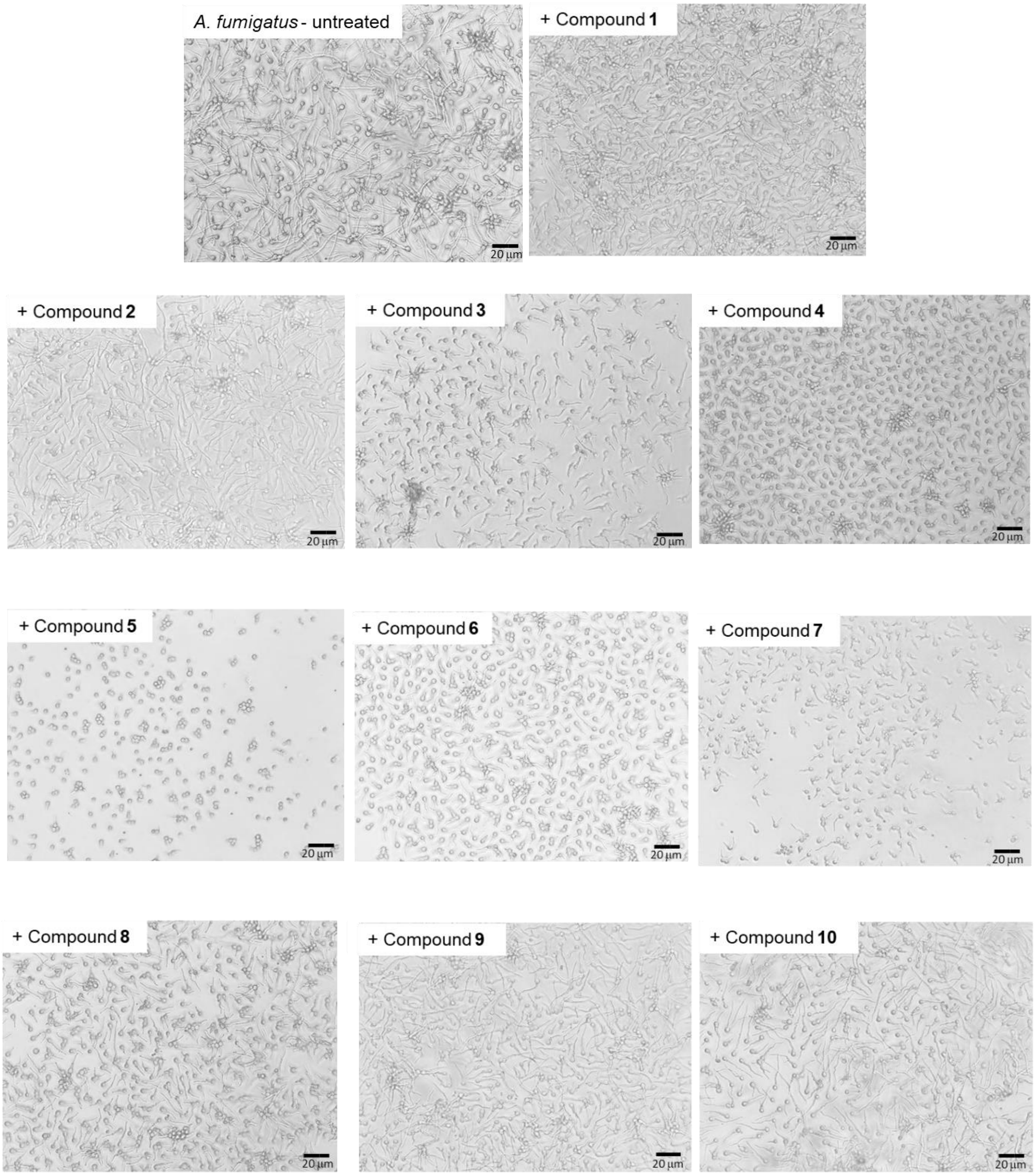

***Figure S 19** A. fumigatus after 24 h incubation with compounds **1-10** (and control) at a concentration of 32 μg·mL^{-1}. Images were taken using an EVOS XL Core Imaging System (ThermoFisher Scientific) and a x40 objective (scale bar = 20 μm).*
